# Structural basis of heterotetrameric assembly and disease mutations in the human *cis*-prenyltransferase complex

**DOI:** 10.1038/s41467-020-18970-z

**Published:** 2020-10-19

**Authors:** Michal Lisnyansky Bar-El, Pavla Vaňková, Adva Yeheskel, Luba Simhaev, Hamutal Engel, Petr Man, Yoni Haitin, Moshe Giladi

**Affiliations:** 1 Department of Physiology and Pharmacology, Sackler Faculty of Medicine, Tel-Aviv University, Tel-Aviv, 6997801 Israel; 2 Institute of Microbiology of the Czech Academy of Sciences, Division BioCeV, Prumyslova, 595, 252 50 Vestec Czech Republic; 3 Department of Biochemistry, Faculty of Science, Charles University, Hlavova 2030/8, 128 43 Prague 2, Czech Republic; 4 Blavatnik Center for Drug Discovery, Tel Aviv University, Tel Aviv, 6997801 Israel; 5 Sagol School of Neuroscience, Tel Aviv University, Tel Aviv, 6997801 Israel; 6 Tel Aviv Sourasky Medical Center, Tel Aviv, 6423906 Israel

**Keywords:** Enzymes, Transferases, Mitochondria

## Abstract

The human *cis*-prenyltransferase (h*cis*-PT) is an enzymatic complex essential for protein N-glycosylation. Synthesizing the precursor of the glycosyl carrier dolichol-phosphate, mutations in h*cis*-PT cause severe human diseases. Here, we reveal that h*cis*-PT exhibits a heterotetrameric assembly in solution, consisting of two catalytic dehydrodolichyl diphosphate synthase (DHDDS) and inactive Nogo-B receptor (NgBR) heterodimers. Importantly, the 2.3 Å crystal structure reveals that the tetramer assembles via the DHDDS C-termini as a dimer-of-heterodimers. Moreover, the distal C-terminus of NgBR transverses across the interface with DHDDS, directly participating in active-site formation and the functional coupling between the subunits. Finally, we explored the functional consequences of disease mutations clustered around the active-site, and in combination with molecular dynamics simulations, we propose a mechanism for h*cis*-PT dysfunction in retinitis pigmentosa. Together, our structure of the h*cis*-PT complex unveils the dolichol synthesis mechanism and its perturbation in disease.

## Abstract (web summary)

The human *cis*-prenyltransferase (h*cis*-PT) complex synthesizes the precursor of the glycosyl carrier dolichol-phosphate and as such it is essential for protein N-glycosylation. The crystal structure of the complex reveals unusual tetrameric architecture and provides insights into dolichol synthesis mechanism and functional consequences of disease-associated h*cis*-PT mutations.

## Introduction

Prenyltransferases are essential enzymes that synthesize isoprenoids, an enormous group of chemically diverse compounds participating in a myriad of cellular processes in all living cells^1^. With chain lengths varying from C_10_ (geranyl diphosphate) to >C_10,000_ (natural rubber), isoprenoids are synthesized by chain elongation of an allylic diphosphate primer via a variable number of condensation reactions with isopentenyl pyrophosphate (IPP, C_5_)^2–4^. Prenyltransferases are classified as *cis*-prenyltransferase or *trans*-prenyltransferase according to the double bonds they form during the condensation reaction^3^. *Cis*-prenyltransferases are further classified according to their product chain length into short-chain (C_15_), medium-chain (C_50–55_), long-chain (C_70–120_), and rubber synthases^1^. Importantly, while short- and medium-chain *cis*-prenyltransferase complexes are homodimeric, long-chain *cis*-prenyltransferases and rubber synthases are formed by a heteromeric subunit assembly of unknown stoichiometry^1,4,5^. To date, only homodimeric enzymes were structurally characterized^6–9^. Therefore, our understanding of the mechanisms allowing long-chain isoprenoid formation by heteromeric enzymes remains limited.

**Fig. 1:**
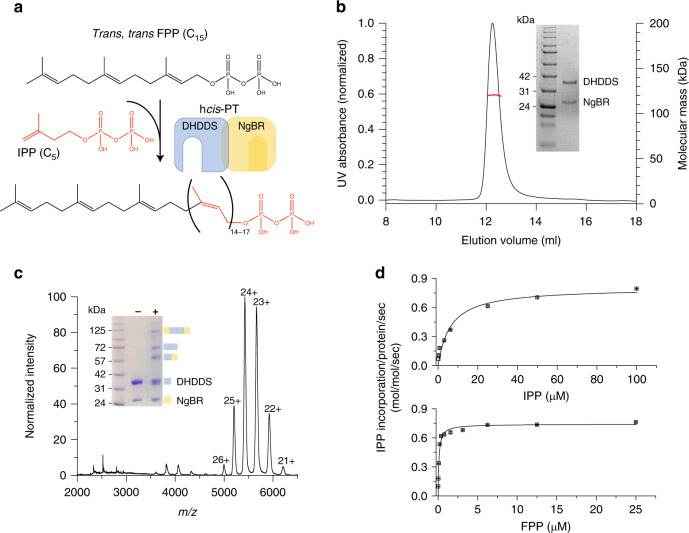
Complex stoichiometry and functional analysis of sh*cis*-PT. **a** Dehydrodolichyl diphosphate synthesis reaction scheme. The IPP moiety is colored red. The h*cis*-PT subunits DHDDS and NgBR are schematically drawn and colored blue and yellow, respectively. **b** Representative SEC-MALS analysis of the purified sh*cis*-PT. The black and red curves indicate UV absorption and molecular mass, respectively. The experiment was performed twice using two different protein batches. Inset: SDS-PAGE analysis of the purified complex. Left lane: molecular weight marker, right lane: purified sh*cis*-PT. Molecular weights (kDa) are indicated. Source data are provided as a Source Data file. **c** Native ESI-MS spectrum obtained using low activation conditions. The main distribution corresponds to the heterotetramer (charge states 21–26). Inset: SDS-PAGE analysis of the complex following glutaraldehyde cross-linking. Left lane: molecular weight marker, middle lane: purified sh*cis*-PT without glutaraldehyde, and right lane: purified sh*cis*-PT with glutaraldehyde. Molecular weights (kDa) are indicated. The experiment was performed twice using two different protein batches. The oligomeric state represented by each state is illustrated using the schematic presentation from panel (**a**). Source data are provided as a Source Data file. **d** In vitro activity of purified sh*cis*-PT assessed as IPP incorporation. Experiments were performed as described in the “Methods” section. Data are presented as mean ± SEM (*n* = 3 independent experiments). Source data are provided as a Source Data file.

The human *cis*-prenyltransferase (h*cis*-PT) complex catalyzes the formation of dehydrodolichyl diphosphate (DHDD, C_85–100_), a long-chain isoprenoid, by chain elongation of farnesyl diphosphate (FPP, C_15_) via multiple condensations with IPP (Fig. 1a)^10^. DHDD is the precursor for dolichol-phosphate, the lipidic glycosyl carrier crucial for N-linked protein glycosylation (Fig. 1a)^11^. Localized to the endoplasmic reticulum, h*cis*-PT is composed of two structurally and functionally distinct subunit types. These include the catalytically active DHDD synthase (DHDDS) and the quiescent Nogo-B receptor (NgBR) subunits^10^. Importantly, while DHDDS subunits are cytosolic, NgBR can be subdivided into an N-terminal transmembrane domain and a C-terminal pseudo *cis*-prenyltransferase domain (Supplementary Fig. 1), which lacks detectable catalytic activity and directly interacts with DHDDS^10^.

In line with the crucial significance of N-linked glycosylation for proper cellular function, mutations in both h*cis*-PT subunits were associated with human diseases. Specifically, DHDDS missense mutations were shown to result in phenotypes ranging from autosomal recessive retinitis pigmentosa (arRP)^12,13^, through developmental epileptic encephalopathies^14^, to a case of fatal congenital disorder of glycosylation reported in a patient heterozygous for both a splice site and a nonsense mutation^15^. Moreover, a missense mutation in the conserved C-terminal RxG motif of NgBR was shown to cause a congenital glycosylation disorder with refractory epilepsy, visual and neurological impairments, congenital scoliosis, and hearing deficit^16^. Finally, recently identified missense mutations in NgBR were shown to contribute to the etiology of Parkinson’s disease^17^. Intriguingly, the different pathogenic mutations in h*cis*-PT seem to have diverse effects on cellular glycosylation. For example, while the missense mutation in the RxG motif of NgBR led to reduced glycosylation in patients fibroblasts^16^, and the patient suffering from the fatal glycosylation disorder displayed hypoglycosylation of serum glycoproteins^15^, the arRP mutation in DHDDS does not seem to have any significant effect on glycosylation in a knock-in mouse model^18,19^, and some patients with DHDDS-related developmental epileptic encephalopathy display normal glycosylation assay results^14^. Thus, the interplay between the genotype and cellular phenotype may be more complex than originally thought and awaits further exploration.

We have previously shown that DHDDS can form functional homodimers, but these complexes exhibit poor catalytic activity compared to the homodimeric orthologs or the heteromeric h*cis*-PT^6,20,21^. Accordingly, previous studies suggested that NgBR can allosterically modulate the activity of the catalytic DHDDS subunit^10,16,21^. Indeed, overexpression of NgBR was shown to significantly enhance h*cis-*PT activity in cells, supporting an NgBR-mediated allosteric modulation of DHDDS activity^10,16^. This effect was suggested to involve a conserved RxG motif, localized to the NgBR C-terminal tail^21^. Recently, the structure of Nus1, the yeast homolog of NgBR, was determined. The structure, devoid of the N-terminal transmembrane domain, revealed that similarly to DHDDS, Nus1 can form homodimers when expressed alone^22^. However, Nus1 does not contain the canonical RxG motif. Instead, it contains a smaller and neutral asparagine residue^22^. Thus, the quest for elucidating the functional and structural roles of NgBR activity in the context of the h*cis*-PT complex is still ongoing and the underlying mechanism remains to be determined.

Despite an immense body of work focusing on the biochemical and structural properties of *cis*-prenyltransferases, our mechanistic understanding of these enzymes arises mainly from investigations of homodimeric prokaryotic, plant, and fungal orthologs^2,4^. Thus, in contrast with the growing clinical relevance of h*cis*-PT, the basic mechanisms underlying its heteromeric assembly, intersubunit communication, and long-chain isoprenoid synthesis remain poorly understood. Recently, the structure of DHDDS in complex with NgBR devoid of its transmembrane region was determined in complex with IPP, and was reported to exhibit a heterodimeric assembly^23^. Here, we established a similar overexpression and co-purification paradigm of the h*cis*-PT complex devoid of its transmembrane region. Unexpectedly, we show that the purified complex formed stable heterotetramers in solution, with equimolar stoichiometry of DHDDS and NgBR subunits. Moreover, it exhibited a marked activity enhancement compared with the purified homodimeric DHDDS^20^. Next, we determined the co-crystal structure of the complex with bound FPP, phosphate, and Mg^2+^ at 2.3 Å resolution. While DHDDS encompasses an active-site reminiscent to that observed in homodimeric family members, the structure exposes the long-sought involvement of NgBR in active-site organization and provides insights into the molecular mechanisms associated with the functional enhancement it confers. Moreover, it reveals the structural organization of the DHDDS C-terminal domain, exhibiting a “helix-turn-helix” motif fold not observed in other *cis*-prenyltransferases, which facilitates complex tetramerization via a dimer-of-heterodimers assembly mode. Importantly, molecular dynamics (MD) simulations pinpoint the mechanisms leading to h*cis*-PT dysfunction in arRP. Finally, the structure lays the foundation toward identifying the determinants governing long-chain isoprenoid synthesis.

## Results

### Purification and in vitro activity characterization of the soluble h*cis*-PT complex

Previously, sequence and biochemical analyses of NgBR revealed that it interacts with DHDDS via its cytosolic C-terminal pseudo *cis*-prenyltransferase homology domain (Supplementary Fig. 1)^10^. Thus, we generated an NgBR construct solely encompassing its cytosolic domain (sNgBR, residues 73^*^–293^*^, where the asterisks designate NgBR residues). Importantly, previous studies using yeast complementation showed that truncation up to position 85^*^ did not affect the ability of NgBR to support cell growth following co-transformation with DHDDS, indicating that the catalytic function of the complex is preserved in the absence of the transmembrane domain^21^. Next, we co-overexpressed the full-length human DHDDS (residues 1–333) and sNgBR in *Escherichia coli*. Following purification, we obtained a homogeneous population of heteromeric soluble h*cis*-PT (sh*cis*-PT) (Fig. 1b).

Intriguingly, during the final size-exclusion purification step, we noticed that the elution volume of sh*cis*-PT corresponds to a higher-than-expected molecular weight range. Size-exclusion chromatography multiangle light-scattering (SEC-MALS) analysis of the purified sh*cis*-PT revealed a monodispersed population with a molecular weight of 119.7 ± 0.4 kDa (Fig. 1b). Within the experimental error of SEC-MALS, this mass may correspond to a stable heterotetramer composed of either two DHDDS (monomer molecular weight = 39.1 kDa) and two sNgBR (monomer molecular weight = 25.2 kDa) subunits or one DHDDS and three sNgBR subunits. In order to determine the stoichiometry of the complex, we used native electrospray ionization (ESI) mass spectrometry (MS) (Fig. 1c). Importantly, native ESI spectra, obtained at low activation conditions to preserve the tertiary structure, revealed a mass of 130.1 ± 0.2 kDa, confirming the heterotetrameric organization of the complex with a stoichiometry of two DHDDS and two sNgBR subunits (Fig. 1c). However, under these conditions, the deconvoluted mass was higher than predicted due to the presence of sodium cations and low-molecular-weight adducts. Indeed, along with partial complex disintegration upon stepwise activation, a shift of the heterotetrameric population toward lower mass (128.7 ± 0.02 kDa) was observed, and an agreement between theoretical and calculated masses was achieved (Supplementary Fig. 2). Finally, we used a cross-linking approach that enables the identification of subunit interactions within the heterotetramer. Treatment with glutaraldehyde, a homobifunctional amine-reactive cross-linker, resulted in the emergence of high-order oligomers, culminating in a heterotetrameric complex with a mass of ~125 kDa (Fig. 1c). In addition, although the cross-linking treatment can theoretically yield three types of dimers (DHDDS homodimer, sNgBR homodimer, and DHDDS–sNgBR heterodimer), we could clearly detect only two bands, corresponding to DHDDS homodimers and DHDDS–sNgBR heterodimers, suggesting that the sNgBR subunits exhibit spatial separation in the context of sh*cis*-PT. Together, the biophysical and biochemical characterizations of sh*cis*-PT suggest a dimer-of-heterodimers arrangement.

Next, in order to validate that the purified complex is catalytically viable, we tested its activity in vitro using a radioligand-based assay (Fig. 1d). The purified sh*cis*-PT exhibited *k*_cat_ = 0.74 ± 0.02 s^−1^ and *K*_M_ = 6.23 ± 1.50 and 0.11 ± 0.01 μM for IPP and FPP, respectively. The *k*_cat_ value is similar to that previously reported for the intact h*cis*-PT^21^ and ~400-fold higher compared to that of homodimeric DHDDS^20^. These results demonstrate that sh*cis*-PT recapitulates the function of the intact complex, further reinforcing that the absence of the N-terminus of NgBR does not impair the catalytic activity of the complex. Moreover, the increased activity of the heteromeric complex compared with homodimeric DHDDS points toward an intersubunit communication mode, since NgBR lacks catalytic activity^1^.

### Structure overview of the sh*cis*-PT subunits

**Fig. 2:**
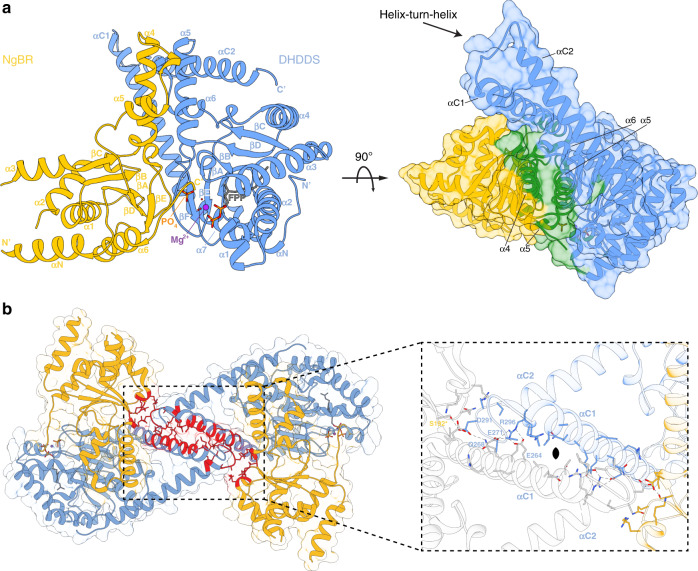
Overall structure of x*cis*-PT. **a** Left: cartoon representation of the ASU, composed of a DHDDS–sNgBRΔ167^*^–175^*^ heterodimer. DHDDS is colored blue and NgBR is colored yellow. Secondary structure elements are labeled. Right: surface representation of the ASU with residues involved in the heterodimeric interface highlighted in green. **b** The heterotetrameric complex assembly mode. Residues involved in tetramerization are shown as sticks and colored red. The rectangle frames a zoom perspective of the interactions between one heterodimer (colored as in panel A) and a heterodimer from the neighboring ASU (colored white).

In order to unveil the structural basis of the functional coupling observed in the context of the sh*cis*-PT, we sought to determine its structure using X-ray crystallography. However, as initial crystallization attempts were unsuccessful, we removed residues 167^*^–175^*^ from NgBR (sNgBRΔ167^*^–175^*^), corresponding to an unresolved loop in the structure of the yeast homolog Nus1^22^, assuming that this region is highly flexible and precludes crystallization. Indeed, this construct (termed hereafter x*cis*-PT) resulted in well-diffracting crystals while demonstrating unperturbed catalytic activity (Supplementary Fig. 3), allowing us to determine its structure in complex with Mg^2+^ and FPP at 2.3 Å resolution (Supplementary Fig. 4). The asymmetric unit (ASU) contains a single heterodimer (Fig. 2a), with Mg^2+^–FPP and an additional phosphate moiety bound only at the DHDDS active-site.

DHDDS can be subdivided into three domains: (i) an N-terminal domain (NTD, residues 1–26), (ii) a canonical catalytic *cis*-prenyltransferase homology domain (residues 27–250), and (iii) a C-terminal domain (CTD, residues 251–333) (Supplementary Fig. 1). The catalytic *cis*-prenyltransferase homology domain, which forms the central region of DHDDS and serves for heterodimerization with NgBR (Fig. 2a, right), is composed of 7 α-helices and 6 β-strands (Supplementary Fig. 1), engulfing an elongated active-site cavity. This domain shares high homology with undecaprenyl diphosphate synthase (UPPS), a bacterial medium-chain *cis*-prenyltransferase homolog, with root-mean-square deviation (RMSD) = 0.76 Å^6^ (Supplementary Figs. 5 and 6, Supplementary Data 1). The NTD and CTD flank the catalytic domain. The NTD is wrapped around the catalytic domain (Fig. 2a, left), with its single α-helix, αN, directly packed against helix α7 via a network of hydrophobic interactions. Last, the CTD, absent from short- or medium-chain *cis*-prenyltransferases, features a “helix-turn-helix” motif composed of two consecutive helices situated immediately downstream of α7 (Fig. 2a, Supplementary Fig. 1). The first helix, αC1, is kinked by 30° relative to the preceding α7 from the catalytic domain. The second helix, αC2, is stabilized against αC1 by a salt bridge between D273 and R306, and numerous electrostatic (E168–K320, E168–R321, D182–R309, and R196–E318) and hydrophobic interactions with the heterodimerization interface between DHDDS and NgBR (Fig. 2a).

Similar to DHDDS, the remnant N-terminal domain of sNgBR (NTD, residues 79^*^–100^*^) also encompasses a single α-helix, αN, likely serving as a structural link between the transmembrane and cytosolic domains in the intact protein. However, while NgBR was previously thought to share the canonical *cis*-prenyltransferase fold, similar to DHDDS^10^, the structure reveals that the two subunits share low structural similarity (RMSD = 2.07 Å, residues 100^*^–293^*^ of sNgBR and 27–250 of DHDDS). Indeed, the pseudo *cis*-prenyltranseferase homology domain of sNgBR encompasses only six α-helices and five β-strands (Fig. 2a, Supplementary Fig. 1), in contrast to the seven α-helices and six β-strands found in the other *cis*-prenyltransferases^2,4^. Moreover, NgBR and its yeast homology, Nus1, do not share high structural similarity (RMSD = 1.33 Å), with the α3 of NgBR occupying the position of the antiparallel βC–βC’ in Nus1 (Supplementary Fig. 5). The described fold of NgBR prevents interactions with substrates and hinders the catalytic activity of NgBR, as specified below.

### Mechanism of tetramerization via a dimer-of-heterodimers assembly

The DHDDS–sNgBR heterodimer is formed through a large interaction interface, with a buried surface area of 1938.0 Å^2^ (Fig. 2a, right). This interface is mainly formed by helices α5, α6, and the βE–βF linker of DHDDS and helices α4, α5, and the βD–βE linker of NgBR, with an architecture reminiscent to that observed in homodimeric *cis*-prenyltransferases^2^. However, the heterodimeric interface also features contacts between the NgBR C-terminus and the active-site of DHDDS. Specifically, the structure reveals that the NgBR C-terminus, which encompasses the RxG motif and was previously suggested to play a critical role in h*cis*-PT activity, extends across the dimerization interface (Fig. 2a, left). This transverse interaction results in its direct involvement in the organization of the active-site of DHDDS and provides a structural framework for intersubunit communication.

While crystal packing analysis using the protein interfaces, surfaces and assemblies server^24^ suggested several possible assemblies, ranging from heterodimers to dodecamers, only one biological tetrameric assembly consistent with the oligomeric state in solution was detected. Importantly, this tetramer is formed by both homotypic interactions between DHDDS “helix-turn-helix” motifs and heterotypic interactions of the “turn” region with sNgBR from adjacent ASUs, burying a total surface area of 793.3 Å^2^ (Fig. 2b). Specifically, this interface is stabilized by two polar networks: (i) a salt-bridge network, centered at the interaction between R296 from the αC2 helix of one heterodimer and E264 and E271 from the  αC1 helix of the adjacent heterodimer, and (ii) a hydrogen-bond network, originating from the interaction of D291, localized to the “turn” between αC1 and αC2, and Q268 from αC1, with S192^*^ of the adjacent heterodimer. Notably, this dimer-of-heterodimer arrangement is consistent with our cross-linking analysis (Fig. 1c).

### Active-site organization

**Fig. 3:**
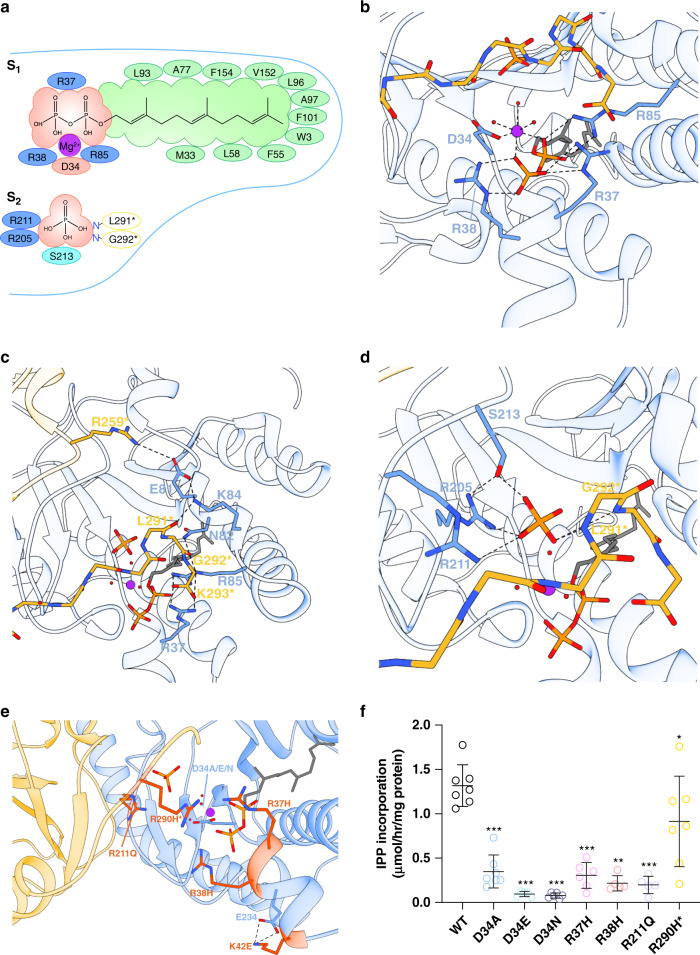
Structural organization of the active-site. **a** Two-dimensional interaction diagram of the S_1_ and S_2_ sites. Hydrophobic interactions, positively, and negatively charged DHDDS residues are colored green, blue, and red, respectively. NgBR residues interacting with the S_2_ site via their backbone nitrogen are indicated in yellow circles. **b** The FPP pyrophosphate-binding region at the S_1_ site. DHDDS is colored blue and NgBR is colored yellow. **c** Interactions of the NgBR C-terminal tail with the pyrophosphate-binding region of the S_1_ site. DHDDS is colored blue and NgBR is colored yellow. **d** Phosphate coordination at the S_2_ site. Mg^2+^ and water molecules are shown as purple and red spheres, respectively. FPP and phosphate are shown as sticks. **e** Mapping of disease-associated mutations onto the x*cis*-PT heterodimer. Disease-associated positions are presented as sticks and colored orange. D34, mutated in this study, is shown as sticks. **f** In vitro activity of purified sh*cis*-PT and mutants thereof was measured as IPP incorporation following 1-h incubation in the presence of 0.1 μM enzyme, 20 μM FPP, and 100 μM IPP. Data are presented as mean ± SEM. *n* = 5 for D34E, R38H, and R211Q, *n* = 6 for D34N and R37H, and *n* = 7 for the WT, D34A, and R290H^*^. One-sided student’s *t* test was performed to compare all the mutants to the WT, except for R38H, for which one-sided Mann–Whitney test was performed. ^*^P = 0.04, ^**^P = 0.0013, ^***^P < 0.001. Source data are provided as a Source Data file.

Although x*cis*-PT was crystallized in the presence of FPP and Mg^2+^, these substrates, along with an additional phosphate molecule, could only be detected in the active-site of DHDDS (Figs. 2a, 3, Supplementary Fig. 4). This active-site is formed by a superficial polar region, stabilizing the interaction with the pyrophosphate headgroups, and a deep hydrophobic tunnel accommodating the elongating carbon chain (Fig. 3a). The active-site contains two substrate-binding sites: an S_1_ site, which binds the initiatory substrate FPP, and an S_2_ site, which binds the IPP molecules used for chain elongation, and is occupied by the phosphate molecule in our structure. Importantly, the C-terminus of NgBR, encompassing the RxG motif, is directly involved in forming the superficial polar region of both sites.

At the S_1_ site, the pyrophosphate moiety of FPP interacts with a Mg^2+^ ion (Fig. 3b, Supplementary Fig. 4). This Mg^2+^ ion, which plays a crucial role in pyrophosphate hydrolysis during the condensation reaction, is octahedrally coordinated by two of the pyrophosphate oxygens, three surrounding water molecules, and one carboxylate oxygen of the strictly conserved D34 (Fig. 3b, Supplementary Figs. 4 and 6, Supplementary Data 1)^2,4^. In accordance with the key role of D34 and the spatial restraints of Mg^2+^ coordination, both the size and charge of aspartate at this position are crucial for catalysis, as substitution to A, E, or N resulted in ~5–20-fold reduction in catalytic activity (1.32 ± 0.09, 0.35 ± 0.07, 0.10 ± 0.01, and 0.08 ± 0.01 μmol/h/mg protein for WT, D34A, D34E, and D34N, respectively; *n* = 5–7, *P* < 0.001 for each mutant) (Fig. 3f, Supplementary Fig. 7). In addition to the interaction with the Mg^2+^ ion, the pyrophosphate moiety is also directly stabilized by R37 and R38 from helix α1, and R85 from the βB–α3 linker (Fig. 3b, Supplementary Fig. 4). Notably, the R37H and R38H mutations were previously associated with developmental epileptic encephalopathy (Fig. 3e). Indeed, these mutations confer an ~5-fold decrease in catalytic activity (0.31 ± 0.06, 0.22 ± 0.04 μmol/h/mg protein for R37H and R38H, respectively; *n* = 6, *P* < 0.001 for R37H, *n* = 5, *P* < 0.01 for R38H), in line with their role in FPP binding (Fig. 3f, Supplementary Figs. 4, 6, and 7, Supplementary Data 1).

While x*cis*-PT was crystallized in the absence of IPP, a phosphate molecule occupying the IPP pyrophosphate group position, as observed in other *cis*-prenyltransferases (Supplementary Fig. 8), is present at the S_2_ site and is stabilized through a concerted coordination by the conserved R205, R211, and S213 (Fig. 3c, Supplementary Figs. 4 and 6, Supplementary Data 1)^2,4,23^. The R211Q mutation was also associated with developmental epileptic encephalopathy (Fig. 3e). As expected, similar to R37H and R38H, R211Q exhibits an ~5-fold reduction in catalytic activity (0.20 ± 0.04 μmol/h/mg protein; *n* = 5, *P* < 0.001) (Fig. 3f, Supplementary Figs. 4 and 7).

Importantly, the structure reveals that the RxG motif critically contributes to the formation of both S_1_ and S_2_ (Supplementary Fig. 4). At S_1_, it stabilizes the βB–α3 linker of DHDDS by forming two polar interaction networks, interweaved between NgBR and DHDDS (Fig. 3c). One network is organized such that two salt-bridge interactions, between E81 and R259^*^, and K84 and the carbonyl oxygen of L291^*^, are anchored together by another salt-bridge interaction between K84 and E81. The other network is formed by G292^*^ backbone carbonyl interaction with N82 and R85, the latter involved in coordination of FPP (Fig. 3b). In addition, K293^*^, forming the C-terminal carboxylate group of NgBR, interacts with both R37 and R85. At S_2_, the backbone nitrogen atoms of L291^*^ and G292^*^ directly coordinate the phosphate molecule (Fig. 3d). Importantly, in addition to the DHDDS-binding site mutants, R290H^*^, localized to the RxG motif, was shown to result in a devastating congenital glycosylation disorder^16^. Situated between the S_1_ and S_2_ sites (Fig. 3e), this residue does not form a direct interaction with either the phosphate molecule or the FPP. However, inspection of other *cis*-prenyltransferase structures, obtained in the absence of Mg^2+^, reveals that this conserved arginine can spatially and electrostatically replace the Mg^2+^ ion, transversely interacting with both the position corresponding to D34 and the pyrophosphate groups at S_1_ and S_2_ (Supplementary Fig. 9). Thus, R290H^*^ results in decreased catalytic activity (0.91 ± 0.19 μmol/h/mg protein; *n* = 7, *P* < 0.05) (Fig. 3f, Supplementary Figs. 4 and 7). Together, our structure reveals the spatial clustering and functional convergence of disease mutations at the active-site, and provides a plausible explanation for the high conservation of the RxG motif due to its role in active-site organization.

### Hydrophobic interactions in the active-site support isoprenoid chain elongation

According to the current model of chain elongation by *cis*-prenyltransferases, the pyrophosphate headgroups are bound at the superficial polar region, while the carbon chains point toward the deep hydrophobic tunnel. During the catalytic cycle, the pyrophosphate group of the initiatory substrate at the S_1_ site (FPP, C_15_) is hydrolyzed, followed by condensation of the remaining carbons with the IPP (C_5_) from the S_2_ site, yielding a 20-carbon polymer. Then, the elongated product translocates to the S_1_ site, where the growing carbon chain permeates deeper into the hydrophobic tunnel of the active-site. Finally, a new IPP molecule binds to the S_2_ site, and the cycle repeats until the active-site can no longer accommodate the long-chain isoprenoid^25,26^.

**Fig. 4:**
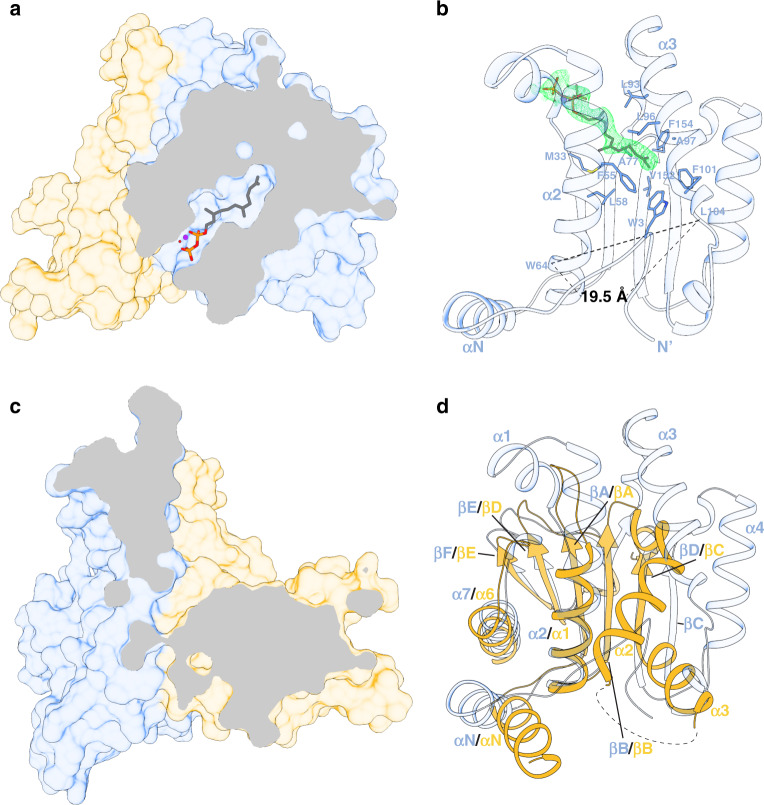
The hydrophobic active-site tunnel. **a** Clipped surface representation of the heterodimer showing the FPP-binding site in DHDDS. DHDDS is colored blue and NgBR is colored yellow. Mg^2+^ and water molecules are shown as purple and red spheres, respectively. FPP is shown as sticks. **b** Coordination of the FPP tail within the active-site. The 2*F*_o_–*F*_c_ electron-density map of FPP, contoured at *σ* = 1, is presented as a green mesh. FPP-interacting residues are shown as sticks. **c** Clipped surface representation of the heterodimer showing the lack of solvent-accessible cavity in NgBR. This view was obtained by superposition of NgBR onto DHDDS as viewed in panel (**a**). **d** Cartoon representation of a superposition between the *cis*-prenyltransferase homology domains of DHDDS and NgBR. The secondary structure elements are indicated. Note the tight packing of strands βΑ, βΒ, and βD, and helices α1 and α6, leading to a collapse of the hydrophobic tunnel in NgBR, prohibiting FPP binding.

The structure reveals that the hydrophobic tunnel of DHDDS is formed by 2 α-helices (α2 and α3) and 4 β-strands (βA, βB, βE, and βF), similar to other *cis*-prenyltransferases^26^ (Fig. 4a, b). It was previously suggested that the opening between α2 and α3 may be larger in DHDDS compared to short- and medium-chain *cis*-prenyltransferases, leading to a larger diameter enabling the accommodation of longer products^26^. Indeed, in DHDDS, the distance between α2 and α3, measured between the C_α_ atoms of W64 and L104, is 19.5 Å (Fig. 4b). In contrast, the distance between the corresponding positions in UPPS (F56 and E96), which synthesizes a 55-carbon isoprenoid, is only 15.3 Å^6^.

Intriguingly, the structure shows that the unique NTD of DHDDS can snake into the binding site, interacting with the bound FPP molecule (Fig. 4b). Moreover, the conserved N-terminal W3 (Supplementary Fig. 6, Supplementary Data 1) interacts with F55, F101, and V152, thereby occluding the outlet of the hydrophobic tunnel of the active-site. This results in a surprisingly small active-site volume of 318 Å^3,27^. Indeed, compared with the 371 Å^3^ in the active-site of the medium-chain UPPS^27^, the x*cis*-PT site is seemingly inadequate for accommodating long-chain products. However, the high B factors of the NTD (Supplementary Fig. 10) suggest that it is mobile, possibly assuming different orientations relative to the active-site under physiological conditions. Indeed, it was suggested that hydrophobic residues (W12, F15, and I19) from helix αN interact with the membrane, resulting in increased catalytic activity in the presence of phospholipids^23^. Thus, we suggest that the NTD may shield the hydrophobic tunnel in the apo or FPP-bound states, while being expelled from the tunnel and interacting with the membrane upon chain elongation.

In addition to the active-site diameter, the composition of its terminal region also plays a key role in determination of product length. In UPPS, L137, localized to the N-terminus of βD, was shown to be vital for determining chain length, with the L137A mutant increasing the product length from C_55_ to C_75_^25^. Our structure reveals that the corresponding position in DHDDS is replaced by C148, a hydrophilic and less bulky residue. Thus, C148 cannot occlude the hydrophobic tunnel outlet as efficiently as L137, similar to the L137A mutant, possibly contributing to long-chain product formation.

NgBR has been long known to lack catalytic activity of its own^10^. Nevertheless, the structural basis for this observation remained obscure. The structure clearly reveals that, in sharp contrast with DHDDS, sNgBR does not contain substrate-binding sites (Fig. 4c). Indeed, while the heterodimerization interface is structurally conserved (Fig. 2a), the NgBR region corresponding to the active-site in *cis*-prenyltransferases displays a significantly different structural arrangement (Fig. 4d). Specifically, strands βΑ, βΒ, and βD and helices α1 and α6 are tightly packed via hydrophobic interactions, leaving this region without a detectable substrate-binding cavity and completely devoid of water molecules. This arrangement provides a structural explanation for the absence of NgBR catalytic activity, due to its incapacity to bind FPP and IPP. Thus, the only active-site of the complex is situated within the *cis*-prenyltransferase homology domain of DHDDS (Fig. 2).

### The molecular mechanism of h*cis*-PT dysfunction in arRP

**Fig. 5:**
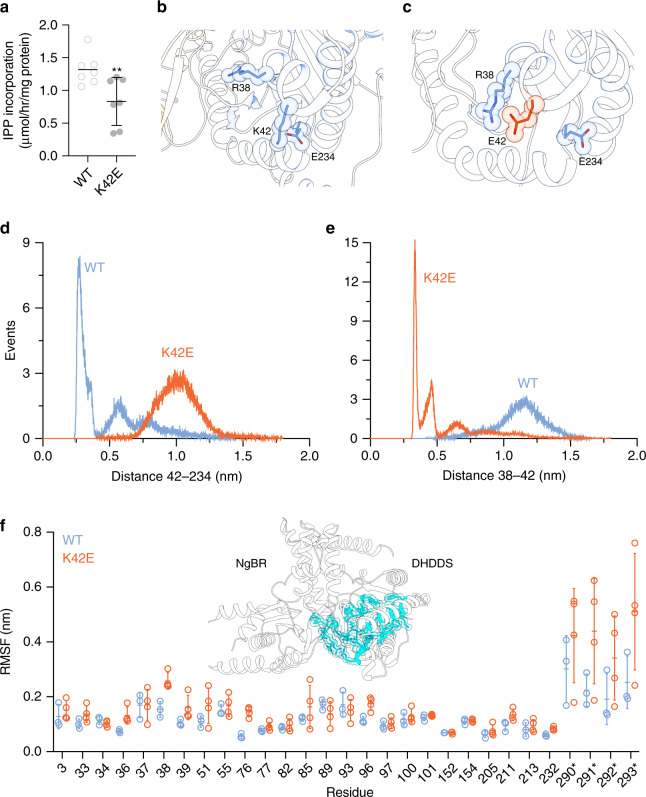
Structural basis for h*cis*-PT-related arRP. **a** In vitro activity of purified sh*cis*-PT harboring the K42E mutation was measured as IPP incorporation following 1-h incubation in the presence of 0.1 μM enzyme, 20 μM FPP, and 100 μM IPP. Data are presented as mean ± SEM, *n* = 7 independent experiments. The activity of the WT is shown for reference. One-sided student’s *t* test was performed for data analysis, ^**^P = 0.0069. Source data are provided as a Source Data file. **b** A representative conformation of the most abundant cluster of the WT protein. R38, K42, and E234 are shown as sticks. R38 points toward the active-site cavity, while K42 and E234 form a stable salt bridge. **c** A representative conformation of the most abundant cluster of the complex harboring DHDDS–K42E. R38, E42, and E234 are shown as sticks. R38 points away from the active-site cavity, forming a new salt bridge with the mutant E42, colored orange. **d** Distance distribution between positions 42 and 234, measured between the charge centers of E234 and K42 (light blue) or E42 (orange). **e** Distance distribution between positions 38 and 42, measured between the charge centers of R38 and K42 (light blue) or E42 (orange). **f** Average (± SD) RMSF of the active-site residues within 5 Å of the crystallized substrates for the WT (light blue, *n* = 3) and mutant (orange, *n* = 4) complexes. Source data are provided as a Source Data file. Inset: Active-site residues within 5 Å of the crystallized substrates are colored cyan and shown as sticks.

Disease mutations in DHHDS are clustered around the pyrophosphate-binding regions of the S_1_ and S_2_ sites (Fig. 3). Based on our structure, the functional perturbation caused by most disease-related positions (R37, R38, and R211) is straightforward, due to their direct involvement in substrate binding. However, the effect of K42E (0.83 ± 0.14 μmol/h/mg protein; *n* = 7, *P* < 0.01) (Figs. 3e and 5a, Supplementary Fig. 7), a mutation leading to isolated retinitis pigmentosa^12,13^, is not as obvious. Careful examination of K42 surroundings revealed that it forms a salt bridge with E234 (Figs. 3e and 5b). Thus, we hypothesized that the functional effect of the K42E mutation is relayed to the active-site via an alternative interaction with adjacent positively charged active-site residues.

To test this hypothesis, we performed an all-atom MD simulation of sh*cis*-PT encompassing DHDDS-WT or DHDDS–K42E (Fig. 5, Supplementary Fig. 11), in the absence of substrates. We calculated three independent 100-ns trajectories for the WT complex and four independent 100-ns trajectories for the mutant complex (Supplementary Fig. 11). Close inspection of the surroundings of position 42 revealed that while the K42–E234 salt bridge remains stable through the simulation (Fig. 5b, d, Supplementary Fig. 11), K42E results in the formation of a new stable salt bridge with the catalytic residue R38 (Fig. 5c, e, Supplementary Fig. 11). Indeed, the distance distributions of positions 42–234 and 38-42 are inversely correlated. Specifically, while the WT protein displays a narrow distribution of short distances between positions 42 and 234, introduction of K42E leads to a wider distribution over longer distances, as expected due to the charge repulsion (Fig. 5d). Conversely, while K42E displays a narrow distribution of short distances between positions 38 and 42, these positions display a wider distribution of longer distances in the context of the WT protein, as expected due to their similar charges (Fig. 5e). Importantly, despite the presence of adjacent positively charged residues (e.g., R37 and K41), E42 forms a stable salt bridge specifically with R38 (Supplementary Fig. 12).

In addition, we examined the effect of K42E on the dynamics of the entire active-site by monitoring active-site residues within 5 Å of the crystallized substrates (Fig. 5f). We noticed that within the timeframe of the simulations, the K42E mutation results in active-site destabilization, including the C-terminus of NgBR, as reflected by the overall increase in RMSD (representing the deviation of the coordinates relative to the initial state of the simulation) in three of four simulations (Supplementary Fig. 11), and by the overall increase in RMSF (representing the fluctuation of the residue side chain relative to its average position along the simulation) (Fig. 5f, Supplementary Fig. 11). These results support the notion that K42E underlies the formation of aberrant polar networks, hindering the capacity of active-site residues to bind substrates, thereby reducing the catalytic activity.

## Discussion

Here, we provide the structure of a heteromeric *cis*-prenyltransferase. Our biochemical and structural analyses of h*cis*-PT reveal a heterotetrameric assembly, formed via a dimer-of-heterodimers mechanism, mainly through homotypic interface formation between the CTD in DHDDS (Fig. 2). Furthermore, the structure elucidates the molecular determinants governing substrate binding, the contribution of the RxG motif of NgBR to active-site formation by transverse interactions with DHDDS, and the effect of active-site resident disease mutations (Fig. 3). In addition, the structure unveils how the architecture of NgBR precludes its endogenous catalytic activity while enabling intersubunit communication, and highlights the molecular determinants supporting formation of long-chain isoprenoid (Fig. 4). Finally, using MD simulations, we unveil the molecular mechanisms of a retinitis pigmentosa causing mutation in h*cis*-PT (Fig. 5).

To date, dimerization is considered as the common tertiary organization of *cis*-prenyltransferases^2,4,8,22^. Additionally, the structurally characterized family members were homodimeric, encompassing an active-site within each subunit, and thus exhibiting an overall functional symmetry. Recently, the presence of a single heterodimer in the crystallographic ASU of h*cis*-PT in complex with IPP was interpreted as an evidence for its heterodimeric stoichiometry^23^. Nevertheless, the elution profile reported is identical to that we present here, suggesting the presence of a common heterotetrameric assembly (Fig. 1). Moreover, we show here using SEC-MALS, native ESI-MS, and cross-linking (Fig. 1) that sh*cis*-PT exhibits a heterotetrameric organization, achieved via a dimer-of-heterodimers assembly mediated by homotypic interactions of the CTD of DHDDS (Fig. 2). Importantly, intact h*cis*-PT harboring the transmembrane domain of NgBR, expressed and purified from Expi293F cells, was previously subjected to size-exclusion analysis. Although its mass was not directly assessed, it also shares a markedly similar elution profile with sh*cis*-PT^21^. This tetrameric assembly mode may contribute to the previously suggested mutual stabilizing effect resulting from NgBR and DHDDS co-expression in cells^10^. Interestingly, a deletion mutant of DHDDS lacking the CTD does not support cell growth in a yeast complementation assay^23^, underscoring the functional significance of tetramerization.

Our structure hints toward the mechanisms allowing long-chain isoprenoid synthesis by h*cis*-PT. While the deep hydrophobic tunnel, engulfing the elongating product, is walled by α2, α3, βA, βB, βE, and βF (Fig. 4), previous studies pinpointed the length of α3 as a key contributor to chain-length determination^26^. Indeed, previous studies of UPPS showed that an insertion of 3 residues, corresponding to ^107^EKE^109^ in human DHDDS, led to an increase in product length from C_55_ to C_70_. The product length was further increased to C_75_ if a 5-residue insertion, mimicking the yeast ortholog Srt1, was introduced, establishing the correlation between the length of α3 and the product^26^. Finally, deletion of the ^107^EKE^109^ sequence was shown to result in product shortening^23^. In full agreement with these observations, the structure of x*cis*-PT reveals that the ^107^EKE^109^ results in a kink in α3, leading to an ~4 Å increase in the hydrophobic tunnel diameter (Fig. 4b) compared with UPPS. In addition to the increased tunnel diameter, we propose that the local environment conferred by C148 may allow expulsion of the elongating product directly into the adjacent membrane during catalysis, allowing the formation of products that exceed the active-site volume. Together, the increased length of α3, the composition of the hydrophobic tunnel outlet, and the membrane association of the complex jointly contribute to long-chain isoprenoid production by h*cis*-PT.

The organization of the CTD of DHDDS, underlying h*cis*-PT tetramerization, provides a mechanistic explanation for the poor activity exhibited by DHDDS homodimers^20^. The helix-turn-helix motif, following α7 (Fig. 2), is incompatible with formation of the extensive interaction network observed within each h*cis*-PT heterodimer active-site (Fig. 3). In contrast, the assembly of DHDDS with NgBR allows the complementation of the active-site by the transverse interactions with the C-terminal tail of NgBR (Fig. 3). Importantly, such transverse interactions are observed also in homodimeric *cis*-prenyltransferases (Supplementary Fig. 9), lacking a C-terminal helix-turn-helix motif, supporting the functional importance of these interactions in coupling active-site organization with enhanced catalytic activity. Consistent with this notion, mutations in the C-terminal tail of NgBR were previously shown to result in reduced catalytic activity^16,21^. Now, our structure offers a mechanistic understanding of the effects imposed by these mutations. Indeed, introduction of R290H^*^ in the context of the intact complex was shown to result in decreased catalytic activity^16,21^. The soluble construct we used for structural investigation also exhibits decreased activity upon mutation (Fig. 3). Structural comparison to other *cis*-prenyltransferases (Supplementary Fig. 9) revealed that the guanidinium group of R290^*^ is necessary and sufficient to substitute the Mg^2+^ ion within the active-site, interacting with both substrates. This interaction may contribute to the translocation of the product pyrophosphate group from S_2_ to S_1_ following the condensation reaction and release of the hydrolyzed pyrophosphate and Mg^2+^ ion^25,26^. In contrast, histidine is both shorter and partially charged under physiological pH, making this residue insufficient to functionally and structurally substitute for the conserved arginine^28^. Moreover, further emphasizing the functional significance of the NgBR C-terminus, previous introduction of the G292A^*^ mutation increased the *K*_M_ for IPP by ~6-fold while reducing the turnover rate by ~12-fold^21^. As shown here, G292^*^ is directly involved in IPP binding (Fig. 3), and measuring the φ/ψ angles reveals that this position can only be occupied by glycine^29^. Finally, we show that the backbone carboxylate of K293^*^ interacts with the catalytic residues R37 and R85 (Fig. 3c). In accordance with the pivotal role of this interaction network, either deletion of K293 or the addition of a terminal alanine resulted in diminished catalytic activity of the intact complex^21^. Together, the heterodimerization architecture observed here supports the notion that although DHDDS is considered as the catalytically active subunit, both subunits are necessary for efficient dolichol synthesis.

By forming the h*cis*-PT complex, DHDDS and NgBR were shown to play a crucial role in cellular dolichol synthesis^10,30^. Mapping disease mutations in DHDDS onto the structure (Figs. 3 and 5) reveals their clustering around the pyrophosphate-binding regions of the S_1_ and S_2_ sites (Fig. 5). Interestingly, these mutations can be subdivided into mutations that directly or indirectly interfere with substrate association. The mutations that directly perturb substrate binding include R37H, R38H, and R211Q (Fig. 3e). These mutations result in a similar reduction in catalytic activity (Fig. 3f) and are clinically associated with developmental epileptic encephalopathies^14^. In contrast, K42E, which leads to isolated arRP^12,13^, is indirectly involved in substrate coordination (Fig. 3e) and leads to a milder reduction in catalytic activity (Fig. 5a). As the pathogenic effect of K42E was not readily apparent from the structure, we hypothesized that the charge reversal caused by K42E may alter the electrostatic interactions that play a vital role in substrate coordination (Figs. 3e and 5). Remarkably, MD simulations uncovered a novel salt bridge between the mutant E42 and the catalytic residue R38 (Fig. 5b–e), hindering substrate binding, and thus providing a mechanistic explanation for the pathogenicity of this mutation (Fig. 5a). Interestingly, additional to this local structural alteration, we also observed a tendency for global increase in active-site dynamics, including the C-terminus of NgBR (Fig. 5f, Supplementary Fig. 11). Inspection of the active-site residues RMSD along the trajectories of the individual simulations revealed destabilization in three of the four replicates (Supplementary Fig. 11c), and the mean RMSF values of these residues are consistently slightly higher in the mutant compared to the WT complex (Fig. 5f). This allosteric effect, emanating from the charge reversal in position 42 and displacement of R38, aligns well with the observation that patients harboring the K42E mutation display a characteristic shortening of their plasma and urinary dolichols^31^. Specifically, we suggest that the globally enhanced dynamics of the active-site may result in weakening of the association with long-chain products, leading to their premature release. Future studies are needed to determine whether altered substrate interaction, either through direct or indirect mechanisms, converges into a similar enhancement of active-site dynamics, resulting in a common outcome of product-length shortening, along with the reduction in catalytic activity (Figs. 3f and 5a).

Together, with the growing spectrum of diseases related to h*cis*-PT dysfunction, the structure sheds light on the mechanisms of dolichol synthesis and their disruption in disease. Moreover, it establishes a molecular framework that may enable the rational design of specific h*cis*-PT activity modulators for the treatment of arRP and additional congenital glycosylation disorders.

## Methods

### Cloning

Full-length human DHDDS (residues 1–333, UniProt Q86SQ9) was cloned into pET-32b plasmid and sNgBR (residues 73^*^–293^*^, UniProt Q96E22) or sNgBRΔ167^*^–175^*^ were cloned into pETM-11 plasmid as thioredoxin (TRX) fusion proteins^32^. The constructs include a 6×His-tag (DHDDS) or Strep-tag (sNgBR/sNgBRΔ167^*^–175^*^) to facilitate protein purification and a TEV-protease (tobacco etch virus) cleavage site to remove the affinity tags and TRX fusion. Mutations were introduced using the QuickChange method and verified by sequencing. Primers used for mutagenesis are listed in Supplementary Table 1.

### Protein expression and purification

*Escherichia coli* T7-expressed competent cells were co-transformed with DHDDS and sNgBR (sh*cis*-PT) or sNgBRΔ167^*^–175^*^ (x*cis*-PT), grown in Terrific Broth medium at 37 °C until reaching OD_600nm_ = 0.6, and induced at 16 °C by adding 0.5 mM isopropyl β-D-1-thiogalactopyranoside (IPTG). Proteins were expressed at 16 °C for 16–20 h, harvested by centrifugation (~5700 × *g* for 15 min), and then resuspended in a lysis buffer containing 20 mM 4-(2-hydroxyethyl)-1-piperazineethanesulfonic acid (HEPES), pH 7.5, 150 mM NaCl, and 1 mM tris(2-carboxyethyl)phosphine (TCEP) and 0.02% (w/v) triton X-100, supplemented with 1 μg/ml DNase I and a protease inhibitor mixture. Resuspended cells were homogenized and disrupted in a microfluidizer. Soluble proteins were recovered by centrifugation at ~ 40,000 × *g* for 45 min at 4 °C. Overexpressed proteins were purified on a HisTrap HP column, followed by purification on a Strep-Tactin column and TEV-protease cleavage of the purification tags and TRX fusions. The reaction mixture was concentrated and loaded onto a Superdex-200 preparative size-exclusion column pre-equilibrated with 20 mM HEPES, pH 7.5, 150 mM NaCl, and 1 mM TCEP. Purified proteins were flash-frozen in liquid nitrogen and stored at −80 °C until use. Protein purity was >95%, as judged by SDS-PAGE.

### Cross-linking

About 8 µM of sh*cis*-PT were cross-linked by incubation with 0.005% glutaraldehyde at room temperature for 15 min. Reactions were quenched by the addition of sodium dodecyl sulfate and β-mercaptoethanol containing sample buffer, followed by 10 min of incubation at room temperature. Cross-linked products were analyzed by SDS-PAGE.

### ESI-MS

sh*cis*-PT (20 µM) was transferred into 200 mM ammonium acetate, pH 7.5, by Zeba Spin columns (0.5 mL, 7-kDa cutoff) and adjusted to 10 µM concentration. The sample was loaded into a homemade quartz-glass ESI tip that was mounted onto a custom-built nESI source interfaced to Waters Synapt G2Si. Analyses at different activation settings were performed. Low activation (trap collision energy 10 V) was used to obtain native-like conditions, while high-trap collisional energies (up to 110 V) were employed to strip the adducts and obtain more accurate mass. Key instrument parameters were sampling cone voltage 40 V, source offset 20 V, trap gas 4 ml/min and source temperature 20 °C, and ESI tip voltage 1.8 kV. Data were analyzed in MassLynx 4.1.

### Enzyme kinetics

The activity of purified sh*cis*-PT was measured using a radioligand-based assay^20,32,33^. About 0.01–0.1 μM of purified proteins were mixed with FPP and [^14^C]-IPP to initiate the reaction in buffer composed of 25 mM Tris-HCl, pH 7.5, 150 mM NaCl, 10 mM β-mercaptoethanol, 0.02% Triton X-100, and 0.5 mM MgCl_2_ at 30 °C. 15 mM EDTA (final concentration) was added to quench the reaction, and 500 µL of water-saturated 1-butanol was added to extract the reaction products by thorough vortexing. Initial rates were measured by quenching the reaction at 10% or lower substrate consumption. The products, encompassing ^14^C, were quantitated using a scintillation counter. The *K*_M_ value of FPP was determined by varying [FPP] while holding [IPP] constant at 100 μM, and the *K*_M_ value of IPP was determined by varying [IPP] while holding [FPP] constant at 10 μM. Kinetic constants were obtained by fitting the data to the Michaelis–Menten equation using Origin 7.0 (OriginLab, USA). For mutant analysis, 0.1 μM of purified proteins were mixed with 20 μM FPP and 100 μM [^14^C]-IPP, and the reaction was quenched after 1 h followed by product extraction and quantitation. The Shapiro–Wilk test of normality was performed using Graphpad Prism 8. Several batches of proteins were tested to ensure that the mutational effect is reproducible compared to the WT. One-sided student’s *t* test and the Mann–Whitney test were used for analysis of normally and non-normally distributed data, respectively, using Graphpad Prism 8.

### Crystallization and structure determination

Initial crystallization screens were performed using ~15 mg/mL purified x*cis*-PT in the presence of 0.5 mM MgCl_2_ and 760 μM FPP at 19 °C using the sitting-drop vapor-diffusion method. Crystals were obtained in 0.1 M NaCl, 0.1 M NaPO_4_, pH 7.0, and 33% w/v PEG 300. Data were collected at 100 K using a wavelength of 0.976 Å at the Diamond Light Source (DLS, Oxfordshire, United Kingdom) beamline I03. Data collection was performed using the Generic Data Acquisition software on-site. Integration, scaling, and merging of the diffraction data were done with the XDS program^34^. The structure was solved by automated molecular replacement and initial model building using the programs MrBump^35^ and CCP4^36^ (Table 1). Iterative model building and refinement were carried out in PHENIX^37^ with manual adjustments using COOT^38^. Ramachandran analysis was performed using MolProbity^39^. About 97.12% and 2.88% of residues were in the Ramachandran favored and allowed regions, respectively. Structural illustrations were prepared with UCSF Chimera (https://www.cgl.ucsf.edu/chimera).

**Table 1:** Data collection and refinement statistics.

	6Z1N: x*cis*-PT
*Data collection*	
Space group	R32:H
*Cell dimensions*	
*a*, *b*, *c* (Å)	184.1, 184.1, 112.6
*α*, *β*, *γ* (°)	90, 90, 120
Resolution (Å)	46.02–2.30 (2.44–2.30)*
*R*_sym_ or *R*_merge_	0.166 (2.026)
*I*/σ*I*	16.3 (1.6)
Completeness (%)	100.0 (100.0)
Redundancy	21.1 (20.7)
*Refinement*	
Resolution (Å)	45.99–2.30
No. of reflections	32,477
*R*_work_/*R*_free_	0.1895/0.2209
*No. of atoms*	
Protein	4,079
Ligand/ion	30
Water	122
*B* factors	56.24
Protein	56.29
Ligand/ion	57.25
Water	54.43
*R.m.s. deviations*	
Bond lengths (Å)	0.006
Bond angles (°)	1.03

Note: Statistics are based on one crystal per dataset.

*Values in parentheses are for the highest-resolution shell.

### Conservation analysis of DHDDS

The Ensembl server^40^ was used to search for human DHDDS orthologs. These sequences were used to generate a multiple-sequence alignment using Clustalw^41^. The resulting alignment was used as input for the Consurf server^42^, which outputs a conservation score for each residue.

### Molecular dynamics (MD)

A single human NgBR–DHDDS heterodimer was used. Mutation K42E was modeled using Schrodinger’s Maestro 11.2. All the structures were prepared using the Protein Preparation Wizard (Schrödinger Release 2017-2: Schrödinger Suite 2019-2 Protein Preparation Wizard; Schrödinger, LLC, New York, NY, 2016) as implemented in Schrodinger’s Maestro 11.2 (Schrödinger Release 2017-2: Schrödinger Suite 2019-2 Protein Preparation Wizard; Schrödinger, LLC, New York, NY, 2016). This protocol adds missing hydrogen atoms considering a pH value of 7.0 ± 1.0, optimizes the hydrogen-bond network, and performs restrained minimization. Crystallographic ligands, ions, and water were removed. The MD simulations were performed using GROMACS version 2018.2^43^, with OPLS forcefield^44^. Each protein complex was submerged in TIP3P water model in a triclinic box with 15 Å extension around the protein. Potassium and chloride ions were added to the water phase in order to neutralize the system and to obtain a salt concentration of 0.15 M. The simulations were conducted in periodic boundary conditions with particle-mesh Ewald electrostatics with 10 Å cutoff for long-range interactions^45^. First, the simulated systems were energy minimized with the steepest descent minimization algorithm in order to remove van der Waals clashes. Afterward, two equilibration steps were used: 100 ps of simulation in NVT ensemble followed by 100 ps of NPT ensemble, where the heavy atoms of the protein are restrained in both steps. During the equilibration stages, an integration time step of 1 fs was used. Finally, the production simulations were carried out for 100 ns with a constant temperature of 300 K under V-rescale coupling algorithm and constant pressure of 1 atm under Parrinello–Rahman coupling algorithm. The LINCS algorithm was applied to bond lengths involving hydrogen, allowing an integration time step of 2 fs^44^. Three and four replicas of MD simulations were conducted for each of the WT and mutant constructs, respectively. The resulting trajectories were visually inspected using VMD 1.9.3 software^46^. Clustering was performed by the clustering analysis tool of Gromacs (gmx cluster). The GROMOS clustering algorithm with a cutoff of 0.12 nm was used to determine the neighboring structures in the clusters. The stability of the resulting trajectories and the average mobility of the protein residues 5 Å around the crystallographic ligand were tested based on the RMSD of the backbone atoms of the protein from the equilibrated structures and on the RMSF, respectively. RMSD and RMSF were calculated using the rms and rmsf utilities of the GROMACS package, respectively. Distances along the trajectories’ time and distances probabilities were calculated using the distance utility.

### Reporting summary

Further information on research design is available in the Nature Research Reporting Summary linked to this article.

## Supplementary information

Supplementary Information

Peer Review File

Supplementary Data 1

Reporting Summary

## Data Availability

Atomic coordinates and structure factors for the structure of x*cis*-PT in complex with Mg^2+^ and FPP have been deposited in the Protein Data Bank with accession number 6Z1N. Source data are provided with this paper.

## Source data

Source Data
