# Supplementary material for: Structural basis of heterotetrameric assembly and disease mutations in the human cis-prenyltransferase complex
Source: Nat Commun. 2020 Oct 19;11:5273. doi: 10.1038/s41467-020-18970-z (PMC7573591; doi:10.1038/s41467-020-18970-z)
Supplement: Supplementary file 1 — Supplementary Information [file 41467_2020_18970_MOESM1_ESM.pdf]

## Supplementary information

**Structural basis of heterotetrameric assembly and disease mutations in the human *cis*-prenyltransferase complex**

Michal Lisnyansky Bar-El[1], Pavla Vaňková[,3], Adva Yeheskel[4], Luba Simhaev[4], Hamutal Engel[4], Petr Man[2], Yoni Haitin[1,5,7,*] and Moshe Giladi[1,6,7,*]

[1] Department of Physiology and Pharmacology, Sackler School of Medicine, Tel-Aviv University, Tel-Aviv, 6997801, Israel

[2] Institute of Microbiology of the Czech Academy of Sciences, Division BioCeV, Prumyslova 595, 252 50 Vestec, Czech Republic

[3] Department of Biochemistry, Faculty of Science, Charles University, Hlavova 2030/8, 128 43 Prague 2, Czech Republic

[4] Blavatnik Center for Drug Discovery, Tel Aviv University, Tel Aviv, 6997801, Israel

[5] Sagol School of Neuroscience, Tel Aviv University, Tel Aviv, 6997801, Israel

[6] Tel Aviv Sourasky Medical Center, Tel Aviv, 6423906, Israel

[7] These authors jointly supervised this work: Yoni Haitin and Moshe Giladi

[*] Correspondence: yhaitin@tauex.tau.ac.il or moshegil@post.tau.ac.il

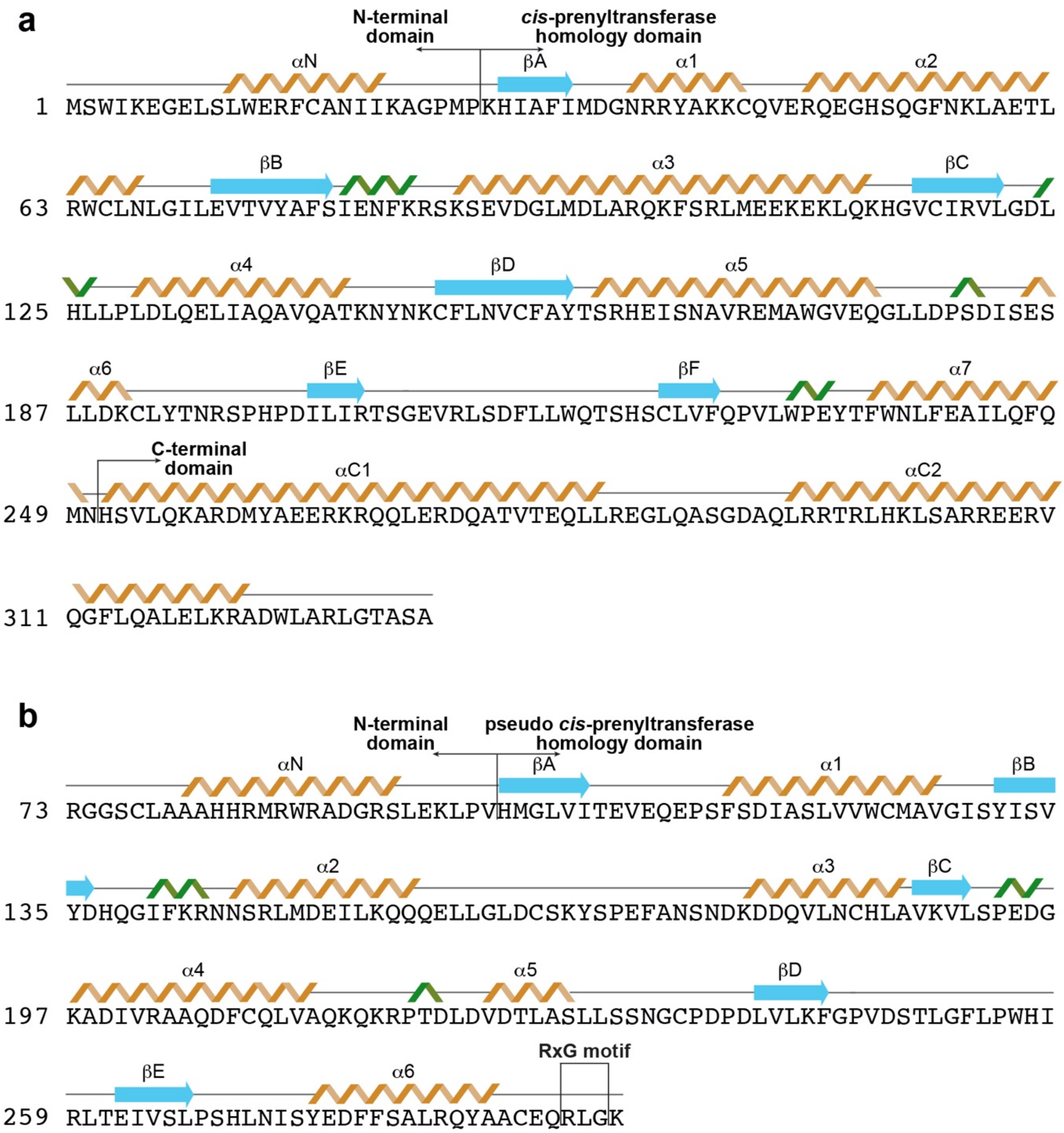


**Supplementary Figure 1. Topology of sh*cis*-PT.** The secondary structures of DHDDS **(a)** and sNgBR **(b)** are indicated above the sequence. The N-terminal domain of DHDDS and NgBR include a single α-helix, αN. The *cis*-prenyltransferase homology domain of DHDDS includes 7 α-helices (α1–7) and 6 β-strands (βA-F), while the pseudo *cis*-prenyltransferase homology domain of NgBR includes 6 α-helices (α1–6) and 5 β-strands (βA-E). Finally, the C-terminal domain of DHDDS includes two α-helices, designated αC1 and αC2, while the C-terminus of NgBR includes the conserved RxG motif.

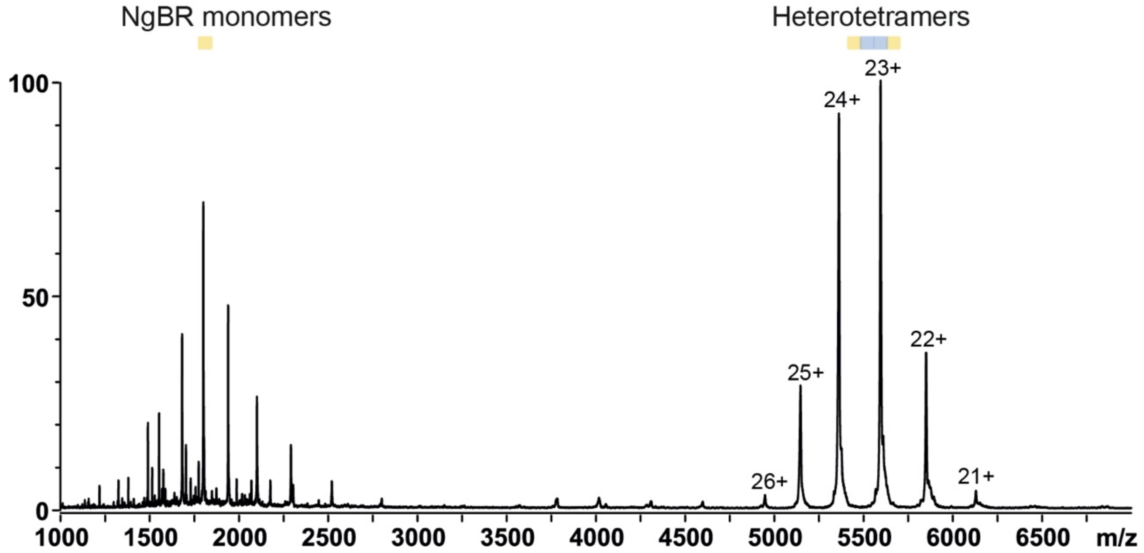


**Supplementary Figure 2. Native ESI analysis of sh*cis*-PT.** Native electrospray ionization (ESI) mass-spectrometry (MS) analysis of sh*cis*-PT under higher collisional activation. Mild activation of sh*cis*-PT led to dissociation of the adducts, providing narrower peaks of the heterotetramer and more accurate mass determination. The calculated mass of the heterotetramer using high activation conditions is 128.7 ± 0.02 kDa. As a side effect of the activation partial complex decomposition can be seen as the presence of lower *m/z* signals corresponding to the individual subunits, specifically NgBR monomers. These peaks are not present if low collisional energy is used (Fig. 1C). Complex subunits, depicting also the stoichiometry, are shown as colored squares (NgBR - yellow, DHDDS - blue) at the top. Charge states of the individual peaks are indicated over the spectrum.

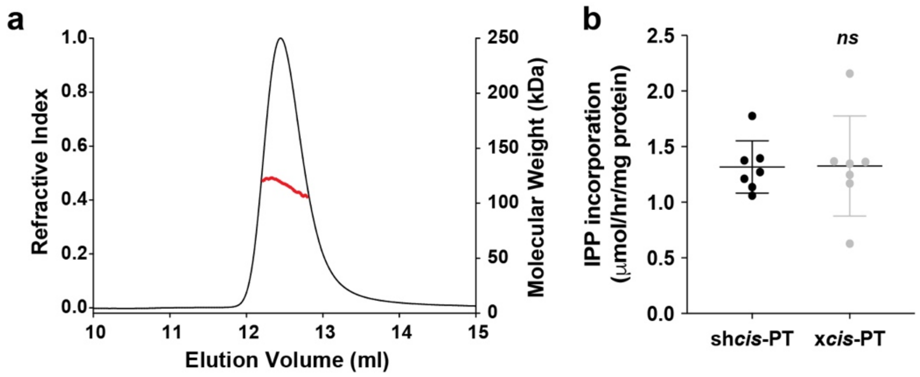


**Supplementary Figure 3. Functional analysis of x*cis*-PT. (a)** SEC-MALS analysis of the purified xh*cis*-PT. The black and red curves indicate refractive index and molecular weight, respectively. **(b)** *cis*-PT activity was measured as IPP incorporation following 1-hour incubation in the presence of 0.1 μM enzyme, 20 μM FPP and 100 μM IPP. Data are presented as mean ± SEM, n = 7 independent experiments. Two-sided student's t-test was performed for data analysis. ns, nonsignificant (P = 0.9684). Source data are provided as a Source Data file.

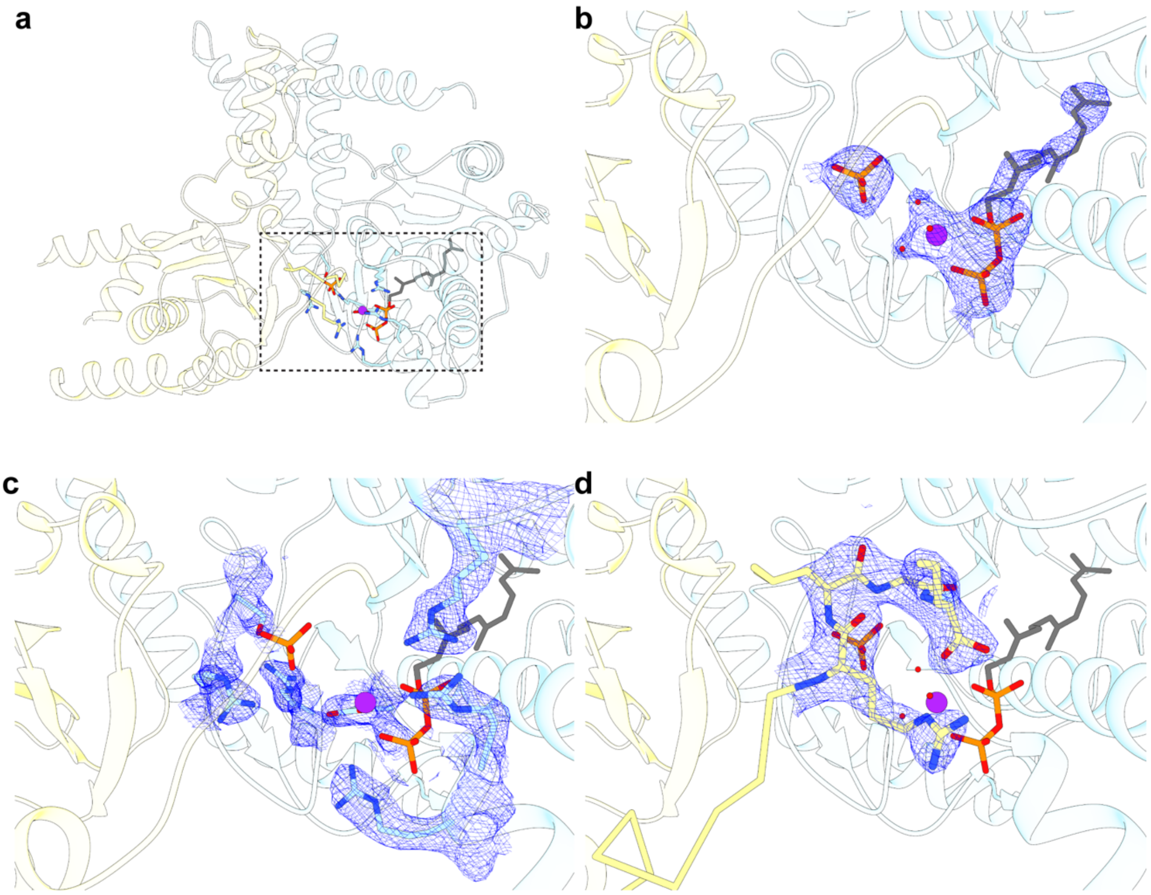


**Supplementary Figure 4. Omit maps of the substrate binding region. (a)** Cartoon representation of the heterodimer. The rectangle frames the substrate binding region shown in panels b-d. DHDDS is colored light blue and NgBR is colored light yellow **(b-d)** m*F*o-D*F*c omit maps calculated by omission of **(b)** substrates (FPP, phosphate, $Mg^{2+}$ and its associated water molecules), **(c)** substrate-coordinating residues (D34, R37, R38, R85, R205, R211, S213) or **(d)** the C-terminus of NgBR (residues 289-293). The maps are shown as blue mesh and contoured at 1.2σ.

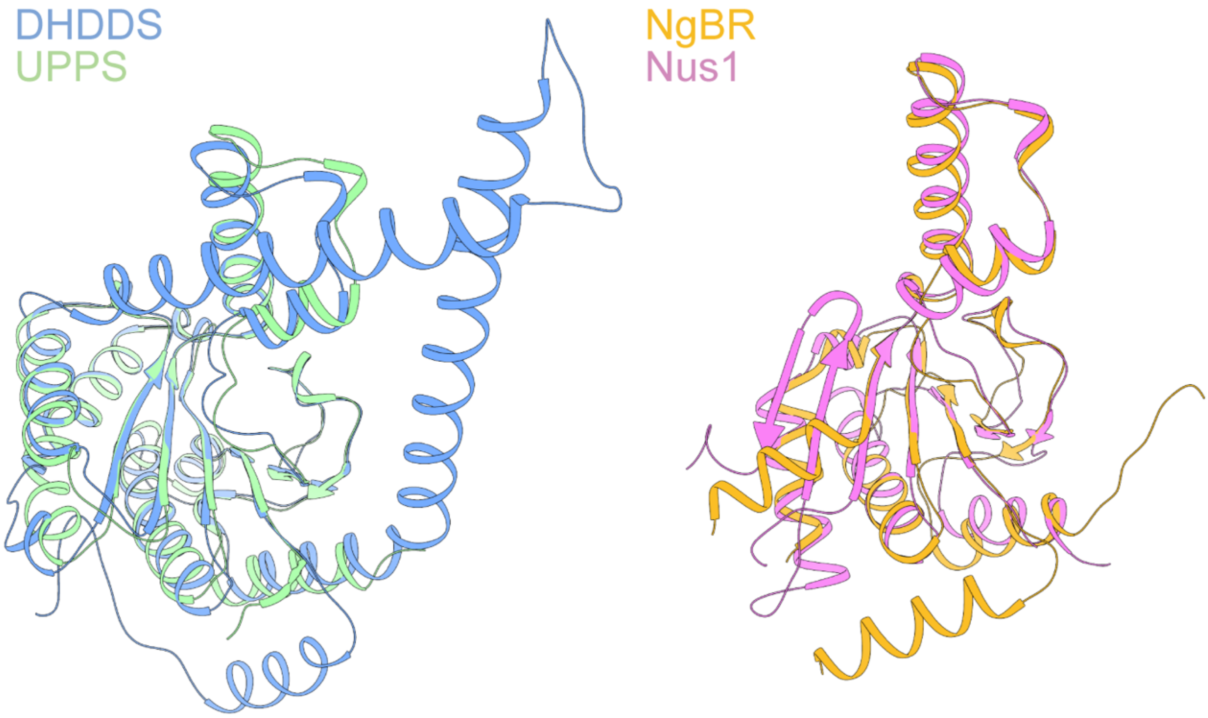


**Supplementary Figure 5. Structural comparison with selected orthologs.** Superposition of DHDDS (blue) and UPPS (PDB 1X06, green) (left) and superposition of NgBR (yellow) and Nus1 (PDB 6JCN, pink) (right). For DHDDS and UPPS, RMSD = 0.76Å while for NgBR and Nus1, RMSD = 1.33Å.

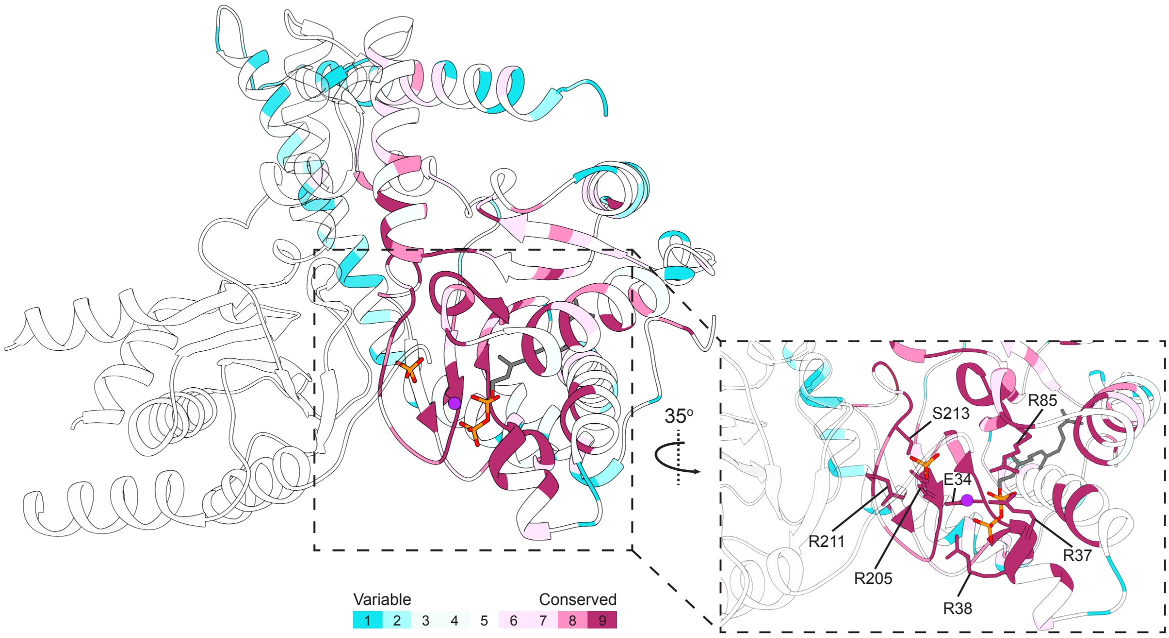


**Supplementary Figure 6. Conservation analysis of DHDDS.** DHDDS colored according to the conservation score of each residue as determined by ConSurf. The rectangle frames the substrate binding site and substrate-coordinating residues are shown as sticks. The conservation score of K42, implicated in autosomal recessive retinitis pigmentosa, is 7.

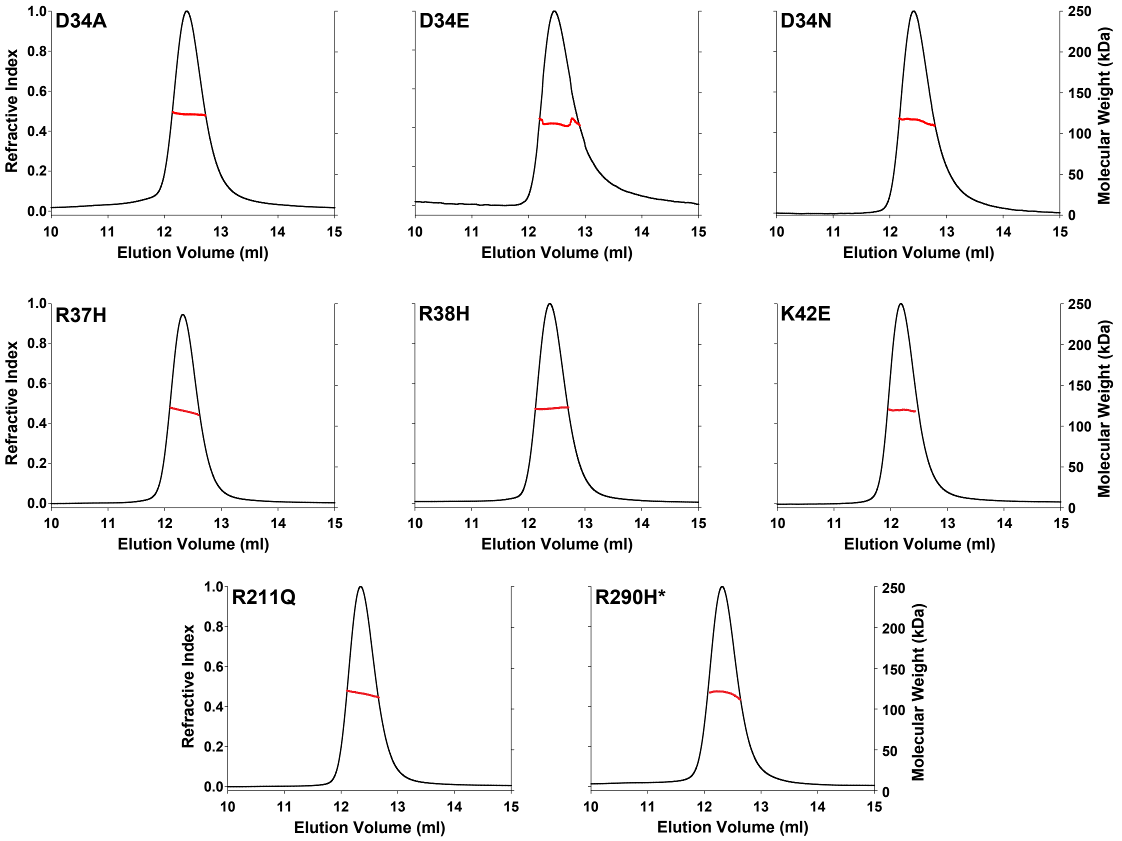


**Supplementary Figure 7. SEC-MALS analysis of sh*cis*-PT mutants.** SEC-MALS analysis of the purified sh*cis*-PT mutants D34A, D34E, D34N, R37H, R38H, K42E, R211Q and R290H*. The black and red curves indicate refractive index and molecular weight, respectively.

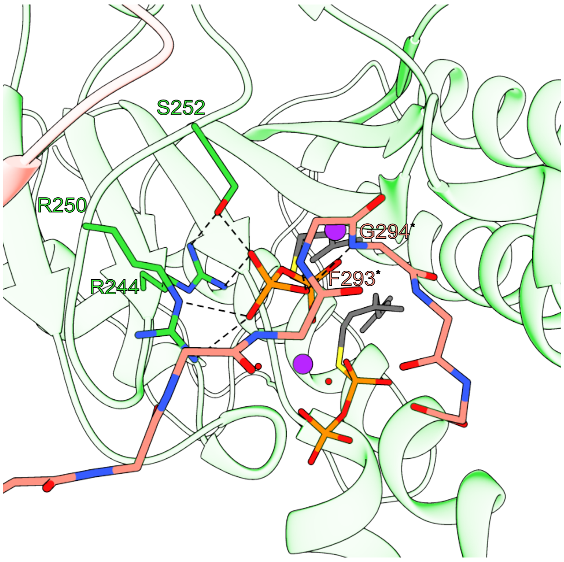


**Supplementary Figure 8. IPP coordination at the $S_2$ site of homodimeric *cis*-prenyltransferase.** The IPP pyrophosphate binding region at the $S_2$ site of the homodimeric *mycobacterium tuberculosis* decaprenyl diphosphate synthase (PDB 6IME). One monomer is colored green and the other pink. IPP and its coordinating residues are shown as sticks. The organization of the binding site and the interaction network with IPP is identical to that observed for the phosphate molecule in the DHDDS active site.

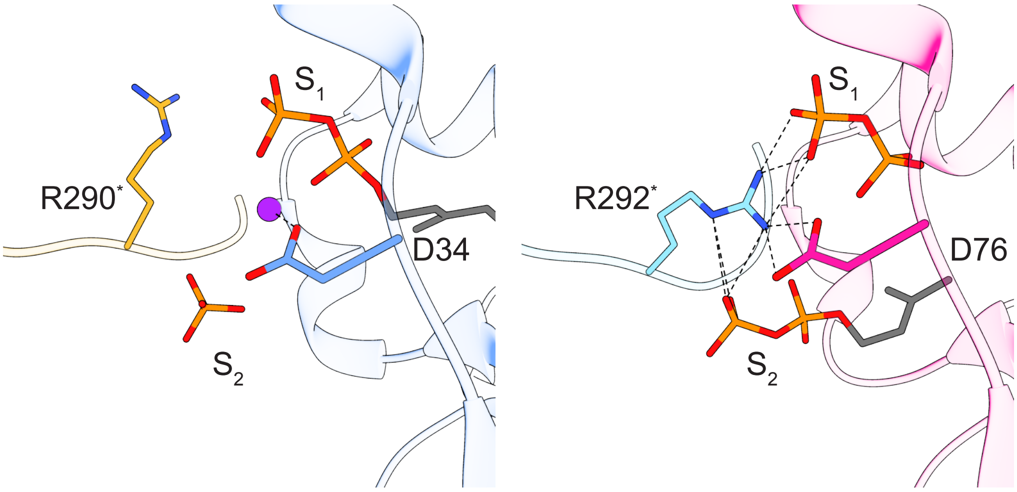


**Supplementary Figure 9. Transverse interactions of R290* with the active site.** Cartoon representation of the pyrophosphate binding region of sh*cis*-PT (left) and the homodimeric *mycobacterium tuberculosis* farnesyl diphosphate synthase (PDB 2VG2) (right), colored by chain. Note the side-chain movement of R292*, corresponding to R290* of NgBR, to replace the $Mg^{2+}$ ion (right).

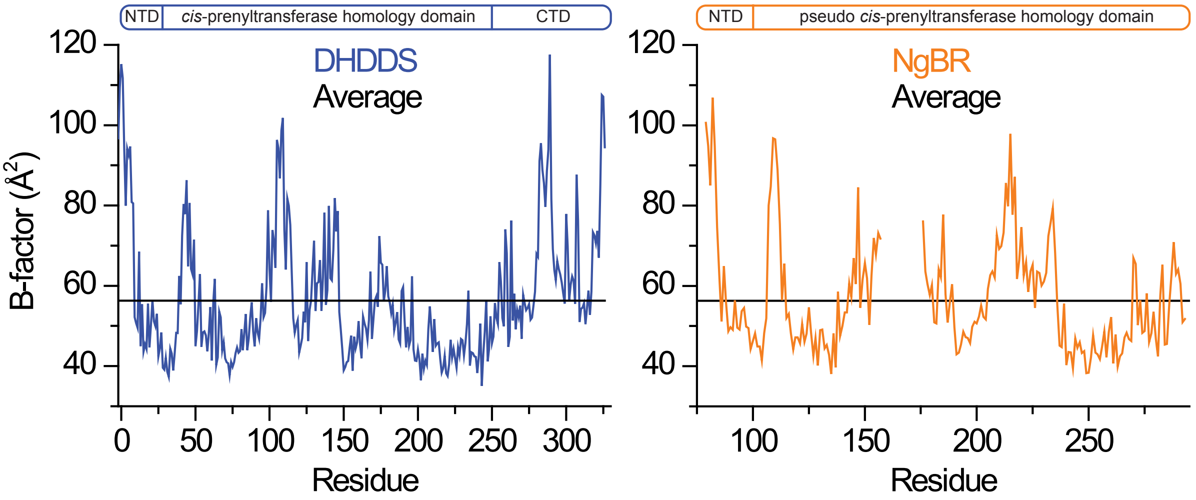


**Supplementary Figure 10. B-factors plots.** Mean residue B-factors of DHDDS (left) and NgBR (right). The black line represents the average macromolecule B-factor.

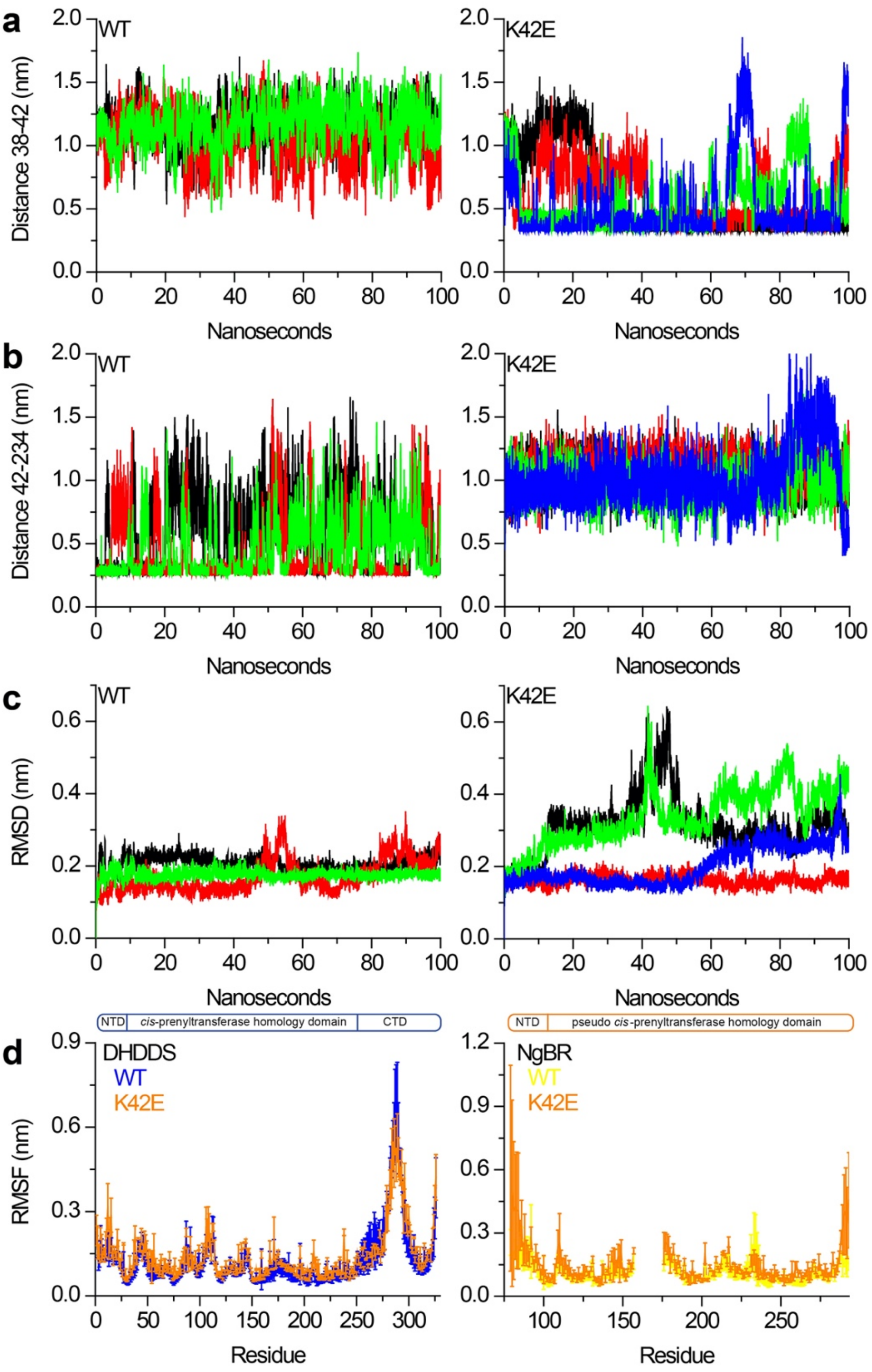


**Supplementary Figure 11. Molecular dynamics analysis of h*cis*-PT. (a)** Distances between the charge centers of R38 and K42 (left) or E42 (right) along the individual simulations trajectories. **(b)** Distances between the charge centers of K42 (left) or E42 (right) and E234 along the individual simulations trajectories. **(c)** RMSD of the active site residues within 5 Å of the crystallized substrates along the individual simulations trajectories for the WT (left) and mutant (right) complexes. **(d)** RMSF of DHDDS (left, n = 3) and NgBR (right, n =4), shown as mean ± SD for each residue. The RMSF curves of the complex harboring the DHDDS-K42E mutation are colored orange.

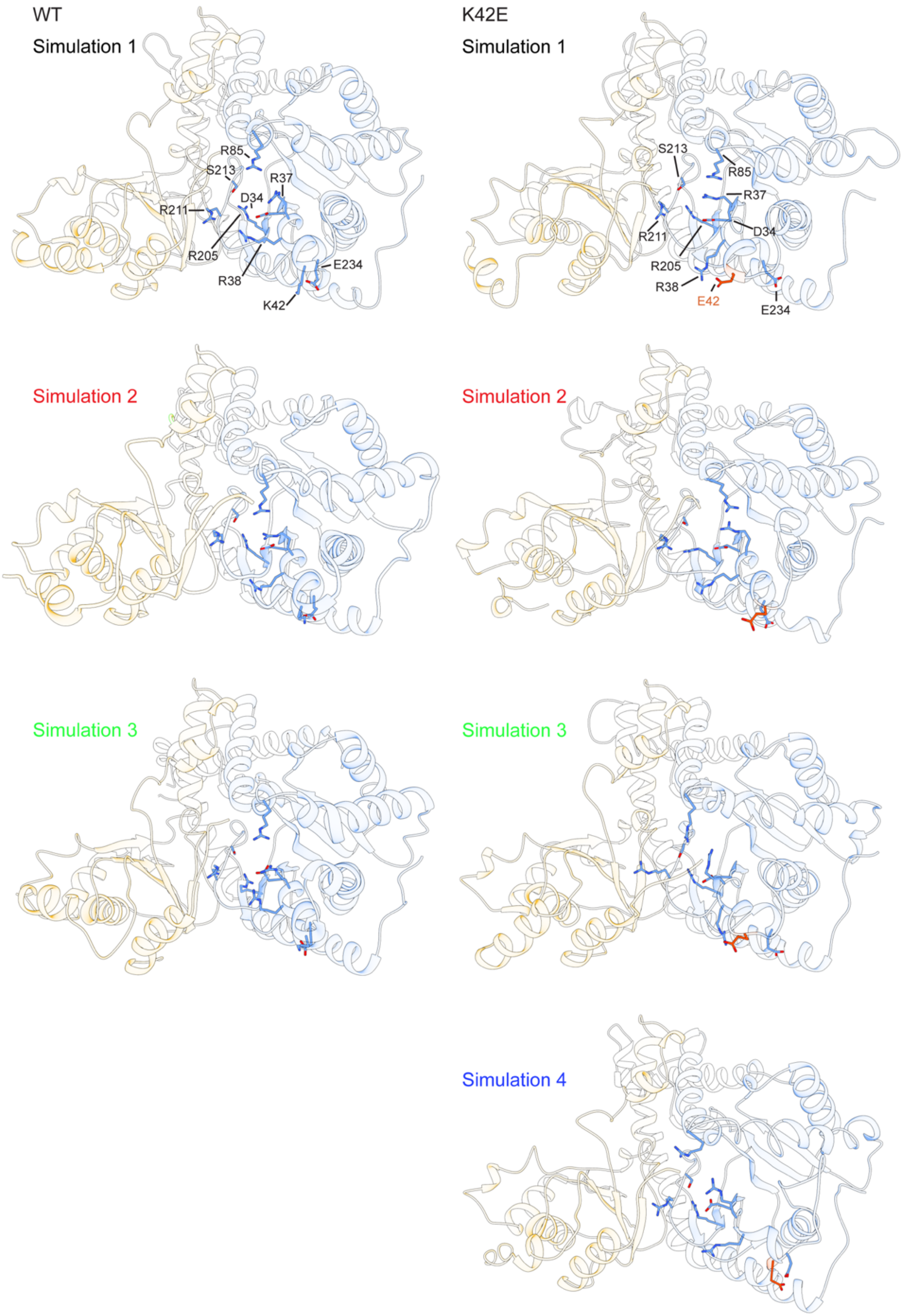


**Supplementary Figure 12. Comparison of the final states from representative molecular dynamics simulations.** The final conformations of apo sh*cis*-PT harboring DHDDS-WT (left) or DHDDS-K42E (right) are shown as cartoon representations. DHDDS is colored blue and NgBR is colored yellow. Substrate coordinating residues are presented as sticks and indicated. The simulations are color coded as in supplementary Fig. 11.

**Supplementary Table 1. PCR primers used for site directed mutagenesis.**

| **Mutation** | **Forward Primer 5'-3'** | **Reverse Primer 5'-3'** |
|---|---|---|
| DHDDS K42E | GCTATGCCAAAGAATGCCAGGTGGAAC | CACCTGGCATTCTTTGGCATAG CGACG |
| DHDDS R37H | GTTTATCATGGATGGTAACCATCGCTATG CCAAAAAATGC | GCATTTTTTGGCATAGCGATGG TTACCATCCATGATAAAC |
| DHDDS R38H | CATGGATGGTAACCGTCACTATGCCAAAA AATGC | GCATTTTTTGGCATAGTGACGG TTACCATCCATG |
| DHDDS R211Q | GAGTGGCGAAGTGCAACTGTCCGATTTTC TG | CAGAAAATCGGACAGTTGCAC TTCGCCACTC |
| DHDDS E234R | GTCCTGTGGCCGAGATATACGTTTTG | CAAAACGTATATCTCGGCCACA GGAC |
| DHDDS D34A | GCGTTTATCATGGCTGGTAACCGTCGC | GCGACGGTTACCAGCCATGATA AACGC |
| DHDDS D34N | CATATTGCGTTTATCATGAATGGTAACCGT CGCTATG | CATAGCGACGGTTACCATTCAT GATAAACGCAATATG |
| DHDDS D34E | GCGTTTATCATGGAGGGTAACCGTCGCT | AGCGACGGTTACCCTCCATGAT AAACGC |
| NgBR R290H | CAGCCTGTGAACAGCACCTGGGAAAGTAA TG | CATTACTTTCCCAGGTGCTGTT CACAGGCTG |
| NgBR Δ167-175 | GACAAAGATGATCAAGTTTTAAATTG | TTTTGAACAATCTAGGCC |
