# Supplementary material for: Structural basis of heterotetrameric assembly and disease mutations in the human cis-prenyltransferase complex
Source: Nat Commun. 2020 Oct 19;11:5273. doi: 10.1038/s41467-020-18970-z (PMC7573591; doi:10.1038/s41467-020-18970-z)
Supplement: Supplementary file 3 — Supplementary Data 1 [file 41467_2020_18970_MOESM3_ESM.docx]

**Supplementary Data 1. Multiple Sequence Alignment of DHDDS orthologs.**

G.lambia ------------------------------------------------------------ 0

E.coli -------------------------------------------------MMLSATQ---- 7

M.luteus --------------------------------------------------MFPIKK---- 6

S.cerevisiae_Srt1 -----MKMPSII-----QIQFVALKRLLVETKEQMCFAVKSIFQRVFAWVMSL---SLFS 47

M.acetivorans -------------------------------------------MKNRTFSMF-YQE---- 12

S.cerevisiae_Rer2 ----------------------------------------METDSGIP-----GHSFVLK 15

c.elegans -----------------------MAANLTADEEDGWF----------------VAQQEQP 21

Drosophila --------------------------------------------------MSWVSDYKYT 10

Zebrafish --------------------------------------------------MSWIREEKLS 10

Cod --------------------------------------------------MSWIREGELN 10

Pike ----------------------------------------MLFHRGCSWKMSWIREGELN 20

Turbot --------------------------------------------------MSWIREGELN 10

eel --------------------------------------------------MSWIREGELN 10

Anole_lizard --------------------------------------------------MSWIREGELS 10

chicken --------------------------------------------------MSWIREGELT 10

Duck --------------------------------------------------MSWIREGELS 10

Beaver --------------------------------------------------MSWIKEGELS 10

Opossum --------------------------------------------------MSWIREGELT 10

Mouse --------------------------------------------------MSWIKEGELS 10

Rat --------------------------------------------------MSWIKEGELS 10

sloth --------------------------------------------------MSWIKEGELS 10

cat --------------------------------------------------MSWIKEGELS 10

Armadilo --------------------------------------------------MSWIKEGELS 10

Elephant --------------------------------------------------MSWIKEGELS 10

Squirrel --------------------------------------------------MSWIKEGELS 10

Dog --------------------------------------------------MSWIKEGELS 10

Cow --------------------------------------------------MSWVKEGELS 10

Goat -----------------------MVLFLVYTELPGWCLIVLCC-FSGARTMSWVKEGELS 36

DHDDS_human --------------------------------------------------MSWIKEGELS 10

Chimpanzee --------------------------------------------------MSWIKEGELS 10

Rabbit --------------------------------------------------MSWIKEGELS 10

Pig --------------------------------------------------MSWIKEGELS 10

Alpaca --------------------------------------------------MSWIKEGELS 10

Horse MLAAVRSASGFLQDSHSYLQALVTSYSLVYPEFSGWFHLVLGSSSVRARTMSWIKEGELS 60

G.lambia ------------------MIPMHVAVIMDGNGRWARKQLQE-RTFGHEQGVSVLESIVDE 41

E.coli ---------PLSEK-LPAHGCRHVAIIMDGNGRWAKKQGKI-RAFGHKAGAKSVRRAVSF 56

M.luteus -----RKAIKNNNI-NAAQIPKHIAIIMDGNGRWAKQKKMP-RIKGHYEGMQTVKKITRY 59

S.cerevisiae_Srt1 WFYVNLQNILIKAL-RVGPVPEHVSFIMDGNRRYAKSRRLP-VKKGHEAGGLTLLTLLYI 105

M.acetivorans -----YEQLLEKEI-LSSEIPDHIAVIMDGNRRYAGQRGRT-RSFGHAMGAEVTEQVIEW 65

S.cerevisiae_Rer2 W----TKNIFSRTLRASNCVPRHVGFIMDGNRRFARKKEMD-VKEGHEAGFVSMSRILEL 70

c.elegans W----WQWLLRRFI-ASGPIPRHVAFVMDGNRRFAKTKHLGNVIKGHEKGFTQLAKILDW 76

Drosophila W----TERIAMRTLRACGYIPHHVAFVMDGNRRFARSQQID-KIEGHSRGFEKLADCLRW 65

Zebrafish L----LERLTANIL-KAGPMPKHVAFIMDGNRRYAQKEHKE-RQEGHTQGFEKLAETLRW 64

Cod L----IERLSANVL-KAGPVPKHVAFIMDGNRRFARKKHVD-RQEGHSQGFNKLAETLRW 64

Pike L----IEKLTANIL-KSGPMPKHVAFIMDGNRRYARKRQVE-RQEGHTQGFDKLAETLRW 74

Turbot L----IEKISANVL-KAGPMPKHVAFIMDGNRRFARKKNMG-RQEGHMQGFNKLAETLRW 64

eel L----IEKLSANVL-KAGPMPKHVAFIMDGNRRFARKKNME-RQEGHMQGFNKLAETLRW 64

Anole_lizard L----LERFCASVI-KAGPMPKHIAFIMDGNRRYAQKHKVK-KLEGHSQGFAKLAQTLQW 64

chicken I----IERFCANII-KAGPMPKHVAFIMDGNRRYAQKCHVE-RQQGHSQGFDKLAQTLRW 64

Duck I----IERFCANII-KAGPMPKHVAFIMDGNRRYAQKCHVE-RQQGHSQGFDKLAQTLRW 64

Beaver L----WERLGVNIL-KAGPVPKHIAFIMDGNRRYAKKCQVQ-KQEGHSQGFNKLAETLRW 64

Opossum L----LERFCANII-KAGPMPKHIAFIMDGNRRYAQKCQVE-RQEGHSQGFDKLAETLRW 64

Mouse L----WERFCANII-KAGPVPKHIAFIMDGNRRYAKKCQVE-RQEGHTQGFNKLAETLRW 64

Rat L----WERFCANII-KVGPVPKHIAFIMDGNRRYAKKCQVE-RQEGHTQGFNKLAETLRW 64

sloth L----WERFCANIL-KAGPVPKHIAFIMDGNRRYAKKCQVE-RQEGHSQGFNKLAETLRW 64

cat L----WERFCANII-KAGPVPKHVAFIMDGNRRYAKKRQVE-RQEGHSQGFNKLAETLRW 64

Armadilo L----WERFCANIL-KAGPMPKHIAFIMDGNRRYAKKCQVE-RQEGHSQGFNKLAETLRW 64

Elephant L----WERFCANII-KAGPMPKHIAFIMDGNRRYAKKCQVE-RQEGHSQGFNKLAETLRW 64

Squirrel L----WERFCANII-KAGPMPKHIAFIMDGNRRYAKKCQVE-RQEGHSQGFNKLAETLRW 64

Dog L----WERFCANII-KAGPMPKHIAFIMDGNRRYAKKCQVE-RQEGHSQGFNKLAETLRW 64

Cow F----WERFCANII-KAGPMPKHIAFIMDGNRRYAKKCQVE-RQEGHSQGFNKLAETLRW 64

Goat F----WERFCANII-KAGPMPKHIAFIMDGNRRYAKKCQVE-RQEGHSQGFNKLAETLRW 90

DHDDS_human L----WERFCANII-KAGPMPKHIAFIMDGNRRYAKKCQVE-RQEGHSQGFNKLAETLRW 64

Chimpanzee L----WERFCANII-KAGPMPKHIAFIMDGNRRYAKKCQVE-RQEGHSQGFNKLAETLRW 64

Rabbit L----WERFCANII-KAGPMPKHIAFIMDGNRRYAKKCQVE-RQEGHSQGFNKLAETLRW 64

Pig F----WERFCANII-KAGPMPKHIAFIMDGNRRYARKCQVE-RQEGHSQGFNKLAETLRW 64

Alpaca F----WERFCANII-KAGPMPKHIAFIMDGNRRYAKKCQVE-RQEGHSQGFNKLAETLRW 64

Horse L----WERFCANII-KAGPMPKHIAFIMDGNRRYAKKCQVE-RQEGHSQGFNKLAETLRW 114

*:..:**** *:* ** *

G.lambia CINCGIRFLTVYAFSTENWSRPPTEVSFLFELLSAAIQRVRTT---Y--RERNVKVQFCG 96

E.coli AANNGIEALTLYAFSSENWNRPAQEVSALMELFVWALDSEVKS---L--HRHNVRLRIIG 111

M.luteus ASDLGVKYLTLYAFSTENWSRPKDEVNYLMKLPGDFLNTFLPE---L--IEKNVKVETIG 114

S.cerevisiae_Srt1 CKRLGVKCVSAYAFSIENFNRPKEEVDTLMNLFTVKLDEFAKRAKDYKDPLYGSKIRIVG 165

M.acetivorans CYEIGVKELTLYAFSTENFQRSEEEVDGLFNLINEKFLKLYNDKRTY--EK-ETQIRVIG 122

S.cerevisiae_Rer2 CYEAGVDTATVFAFSIENFKRSSREVESLMTLARERIRQITERGELA--CKYGVRIKIIG 128

c.elegans CNRFGIREITVYAFSIENFKRSEEEVSGLMRLAEEKFQKLLNDSEKL--DEKRICFRFYG 134

Drosophila CLDVGVREVTTFAFSIENFKRSNEEVEGLFNLAREKFARLLEETARL--DEHGIRIRVIG 123

Zebrafish CLNLGIHEVTVYAFSIENFKRSKEEVDGLMELARQKFSRLLTEQENL--EKHGVCIRVLG 122

Cod CLQLDIPEVTVYAFSIENFNRSREEVDGLMELAKLKFLRLLDERDKL--EKHGVCIRVLG 122

Pike CLNLNILEVTVYAFSIENFKRSKDEVDGLMELAKQKFIKLLEEQENL--EKHGVCIRVLG 132

Turbot CKHLNIPEVTVYAFSIENFKRTKDEVDGLMELAKQKFERLLEERDNL--EKHGVCIRVLG 122

eel CKHLDIQEVTVYAFSIENFKRPKDEVDGLMELARQKFERLLEEHENL--EKHGVCIRVLG 122

Anole_lizard CLSLGVREVTVYAFSIENFKRPREEVDGLMQLAREKFTRLLEEQENL--EKHGVCVRILG 122

chicken CLNLGIREVTVYAFSIENFKRSKEEVDGLMDLARQKFSRLLEEQENL--KKHGVCIRVLG 122

Duck CLNLGIREVTVYAFSIENFKRSKEEVDGLMDLARQKFSRLLEEQEKL--KKHGVCIRVLG 122

Beaver CLHLGIPEVTVYAFSIENFKRPKNEVDGLLDLTRNKLQRLMEEQEKL--KKYGVCVRVLG 122

Opossum CLNLGIREVTVYAFSIENFKRSKNEVDGLMELARQKFTRLMEEQEKL--EKHGVCIRVLG 122

Mouse CLNLGILEVTVYAFSIENFKRSKSEVDGLLDLARQKFSCLMEEQEKL--QKHGVCIRVLG 122

Rat CLNLGILEVTVYAFSIENFKRSKSEVDGLLDLARQKFSCLMEEQEKL--KKHGVCIRVLG 122

sloth CLNLGIMEVTVYAFSIENFKRSKSEVDGLMDLAREKFSRLMEEQEKL--QKHGVCIRVLG 122

cat CLNLGILEVTVYAFSIENFKRSKSEVDGLMDLARQKFSRLMEEQEKL--QKHGVCIRVLG 122

Armadilo CLNLGIMEVTVYAFSIENFKRSKSEVDGLMDLARQKFSRLMEEQEKL--QKHGVCIRVLG 122

Elephant CLNLGILEVTVYAFSIENFKRSKSEVDGLMDLARQKFSRLMEEQEKL--QKHGVCIRVLG 122

Squirrel CLNLGILEVTVYAFSIENFKRSKSEVDGLLDLARQKFTCLMEEQEKL--QKHGVCIRVLG 122

Dog CLNLGILEVTVYAFSIENFKRSKSEVDGLMDLARQKFSRLMEEQEKL--QKHGVCIRVLG 122

Cow CLNLGILEVTVYAFSIENFKRSKSEVDGLMDLAREKFSRLMEEQEKL--QKHGVCIRVLG 122

Goat CLNLGILEVTVYAFSIENFKRSKSEVDGLMDLAREKFSRLMEEQEKL--QKHGVCIRVLG 148

DHDDS_human CLNLGILEVTVYAFSIENFKRSKSEVDGLMDLARQKFSRLMEEKEKL--QKHGVCIRVLG 122

Chimpanzee CLNLGILEVTVYAFSIENFKRSKSEVDGLMDLARQKFSRLMEEQEKL--QKHGVCIRVLG 122

Rabbit CLNLGILEVTVYAFSIENFKRSKSEVDGLMDLARQKFSRLMEEQEKL--QKHGVCIRVLG 122

Pig CLNLGVLEVTVYAFSIENFKRSKSEVDGLMDLARQKFSRLMEEQEKL--QKHGVCIRVLG 122

Alpaca CLNLGVLEVTVYAFSIENFKRSKGEVDGLMDLARQKFSRLMEEQEKL--QKHGVCIRVLG 122

Horse CLNLGVLEVTVYAFSIENFKRSKSEVDGLMDLARQKFSRLMEEQEKL--QKHGVCIRVLG 172

. .: : :*** **:.* **. *: * : .. *

G.lambia ERTTQIPETVIAAMNCIEQDTAACTGLILSVCFNYGGHTEIAQACRSVLADCLEGDAVEN 156

E.coli DTS-RFNSRLQERIRKSEALTAGNTGLTLNIAANYGGRWDIVQGVRQLAEKVQQGNLQPD 170

M.luteus FID-DLPDHTKKAVLEAKEKTKHNTGLTLVFALNYGGRKEIISAVQLIAERYKSGEISLD 173

S.cerevisiae_Srt1 DQS-LLSPEMRKKIKKVEEITQDGDDFTLFICFPYTSRNDMLHTIRDSVEDHLENKS--P 222

M.acetivorans DRT-KLPAFLNKSIEKIEKATETHRKFNLNVAIAYGGRQDIMQAVRDIAACVSSGKLSLE 181

S.cerevisiae_Rer2 DLS-LLDKSLLEDVRVAVETTKNNKRATLNICFPYTGREEILHAMKETIVQHKKG----A 183

c.elegans NRS-LLSSRLQKLMSDIEHRTENFDGGRLNVCMPYTSRDEIARSFETIRKHVKDGKVNVD 193

Drosophila NIE-LLPHDLQKLVASAMLSTERNDKLFLNVAFAYTSRDEITQAVETILRHGSQD-LAGE 181

Zebrafish DLT-LLPEDLQTLIAKAVVSTRAHNKCFLNVCFAYTSRHEIANAVKEMAWGVEQGLIKSS 181

Cod DLT-LLPLDLQQLIAKAVVATREHNRCFLNVCFAYTSRHEMANAVREMAWGVEQGLIKAS 181

Pike DLT-LLPLDLQQHIARAVVATRSHNKCFLNVCFAYTSRHEIANAVREMAWGVEQGHIKSS 191

Turbot DLN-MLPLDLQQVIAKAVLTTRVHNKCFLNVCFSYTSRYEITNAVREMAWGVEQGLIKAS 181

eel DLN-MLPLDLQKLIAKAVVTTRSHNKCFLNVCFAYTSRYEITNAVREMAWGVEQGLIRAS 181

Anole_lizard DLP-LLPRDIQELIAKVVLATKQYNTCFLNIAFAYTSREEISNAVKELAWGVEEGLLQPS 181

chicken DLP-LLPLDVQELIAQAVMATRNYNKCFLNVCFAYTSRHEISNAVREMAWGVEQGLLEPS 181

Duck DLP-LLPLDIQELIAQAVLATRNYNKCFLNVCFAYTSRHEISNAVREMAWGVEQGLLEPS 181

Beaver DLH-LLPLDLQELIAQGVQATKTYNRCFLNICFAYTSRHEITNAVREMAWGVEQGLLDPS 181

Opossum DLQ-LLPLDLQELIAKAVQATHNYSKCFLNVCFAYTSRHEISNAVKEMAWGVEQGLLDPS 181

Mouse DLH-LLPLDLQEKIAHAIQATKNYNKCFLNVCFAYTSRHEIANAVREMAWGVEQGLLEPS 181

Rat DLH-LLPLDLQKKIAQAVQATKNYNKCFLNVCFAYTSRHEITNAVREMAWGVEQGLLEPS 181

sloth DLH-LLPLDLQELIAQAVQATKNYNKCFLNVCFAYTSRHEISNAVREMAWGVEEGLLDPS 181

cat DLH-LLPLDLQELIAQAVQATKNYNKCFLNVCFAYTSRHEISNAVREMAWGVEQGLLDPS 181

Armadilo DLH-LLPLDLQELIAQAVQATRNYNKCFLNVCFAYTSRHEISNAVREMAWGVEEGLLDPS 181

Elephant DLH-LLPLDLQELIAQAVEATRNYNKCFLNVCFAYTSRHEISNAVREMAWGVEQGLLDPS 181

Squirrel DLH-LLPLDLQELIAQAVQATKNYNKCFLNVCFAYTSRHEITNAVREMAWGVEQGLLDPS 181

Dog DLH-LLPLDLQELIAQAVRATKHYNKCFLNVCFAYTSRHEISNAVREMAWGVEQGLLDPS 181

Cow DLH-LLPLDLQELVAQAVQTTKNYNKCFLNVCFAYTSRHEISNAVREMAWGVEQGLLDPS 181

Goat DLH-LLPLDLQELVAQAVQTTRNYNKCFLNICFAYTSRHEISNAVREMAWGVEQGLLDPS 207

DHDDS_human DLH-LLPLDLQELIAQAVQATKNYNKCFLNVCFAYTSRHEISNAVREMAWGVEQGLLDPS 181

Chimpanzee DLH-LLPLDLQELIAQAVQATKNYNKCFLNVCFAYTSRHEISNAVREMAWGVEQGLLDPS 181

Rabbit DLH-LLPLDLQELIAQAVRATKNYNKCFLNVCFAYTSRHEISNAVKEMAWGVEQGLLDPS 181

Pig DLH-LLPLDLQELIAQAVQATKNYNRCFLNVCFAYTSRHEISNAVREMAWGVEQGLLDPS 181

Alpaca DLH-LLPLDLQELIAQAVQATKNYNKCFLNVCFAYTSRHEISNAVREMAWGVEQGLLDPS 181

Horse DLH-LLPLDLQELIAQAVQATKNYNKCFLNVCFAYTSRHEISNAVREMAWGVEQGLLDPS 231

: : * * .. * .: :: . ..

G.lambia IKTRLQMPIEQFIQQIDTHLYAN---LPPVDLLIRTGCEKRLSNFLPWHLAYAEI--IFS 211

E.coli QI-----DEEMLN---QHVCMHE---LAPVDLVIRTGGEHRISNFLLWQIAYAEL--YFT 217

M.luteus EI-----SETHFN---EYLFTAN---MPDPELLIRTSGEERLSNFLIWQCSYSEF--VFI 220

S.cerevisiae_Srt1 RI-----NIRKFT---NKMYM--GFHSNKCELLIRTSGHRRLSDYMLWQVHENA-TIEFS 271

M.acetivorans DV-----DENLIS---KHLYPAPGVSVPNVDLIVRTGGDERVSNFLPWQANGSECATYFC 233

S.cerevisiae_Rer2 AI-----DESTLE---SHLYTAG---VPPLDLLIRTSGVSRLSDFLIWQASSKGVRIELL 232

c.elegans EI-----NESMID---ACLDSGCG--GTSPDLFIRTSGEHRLSDFLMWQASETHV--YFD 241

Drosophila DI-----SERLLE---ECLYTRH---SPPPDLVFRTSGETRLSDFMMWQLSTSVL--YFS 228

Zebrafish DV-----SEVLLS---ECLYSSN---SPNPDLLIRTSGEVRLSDFLLWQTSYSCL--VFQ 228

Cod DV-----SEALLS---ECLYSSN---SPNPDLLIRTSGEVRLSDFLLWQTSHSCL--VFQ 228

Pike DV-----SEALLS---QCLYSSN---SPNPDLLIRTSGEVRLSDFLLWQTSHSCL--VFQ 238

Turbot DV-----SEPLLS---ECLYSNN---SPNPDLLIRTSGEVRLSDFLLWQTSHSCL--VFQ 228

eel DV-----SEVLLS---DCLYSNN---SPNPDLLIRTSGEVRLSDFLLWQSSHSCI--VFQ 228

Anole_lizard DV-----SESLID---KCLYSSK---SPHPDILIRTSGEVRLSDFMLWQTSHSCL--VFQ 228

chicken DV-----SESLLD---KCLYTSN---SPDPDLLIRTSGEVRLSDFLLWQTSHSCL--VFQ 228

Duck DV-----SESLLD---KCLYTSN---SPDPDLLIRTSGEVRLSDFLLWQTSHSCL--VFQ 228

Beaver DV-----SESLLD---KCLYSNH---SPNPDLLIRTSGEVRLSDFLLWQASHSCL--VFQ 228

Opossum DV-----SESLLD---KCLYTNN---SPNPDILIRTSGEVRLSDFLLWQTSHSCL--VFQ 228

Mouse DV-----SESLLD---KCLYSNH---SPHPDILIRTSGEVRLSDFLLWQTSHSCL--VFQ 228

Rat DV-----SESLLD---QCLYSNH---SPQPDILIRTSGEVRLSDFLLWQTSHSCL--VFQ 228

sloth DI-----SESLLD---KCLYTSC---SPHPDILIRTSGEVRLSDFLLWQTSHTCL--VFQ 228

cat DI-----SESLLD---KCLYTNH---SPHPDILIRTSGEVRLSDFLLWQTSHSCL--VFQ 228

Armadilo DV-----SESLLD---KCLYTNH---SPHPDILIRTSGEVRLSDFLLWQTSHTCL--VFQ 228

Elephant DI-----SESLLD---KCLYTNH---SPHPDILIRTSGEVRLSDFLLWQTSHSCL--VFQ 228

Squirrel DI-----SESLLD---KCLYTNH---SPHPDILIRTSGEVRLSDFLLWQTSHSCL--VFQ 228

Dog DV-----SESLLD---KCLYTNH---SPHPDILIRTSGEVRLSDFLLWQTSHCCL--VFQ 228

Cow DV-----SESLLD---KCLYTNH---SPHPDILIRTSGEVRLSDFLLWQTSHSCL--VFQ 228

Goat DV-----SESLLD---KCLYTNH---SPHPDILIRTSGEVRLSDFLLWQTSHSCL--VFQ 254

DHDDS_human DI-----SESLLD---KCLYTNR---SPHPDILIRTSGEVRLSDFLLWQTSHSCL--VFQ 228

Chimpanzee DI-----SESLLD---KCLYTNR---SPHPDILIRTSGEVRLSDFLLWQTSHSCL--VFQ 228

Rabbit DV-----SESLLD---KCLYTSH---SPHPDILIRTSGEVRLSDFLLWQTSHSCL--VFQ 228

Pig DV-----SESLLD---KCLYTNH---SPHPDILIRTSGEVRLSDFLLWQTSHSCL--VFQ 228

Alpaca DV-----SESLLD---KCLYTNH---SPHPDILIRTSGEVRLSDFLLWQTSHSCL--VFQ 228

Horse DV-----SESLLD---KCLYTNH---SPHPDILIRTSGEVRLSDFLLWQTSHSCL--VFQ 278

: ::..**. *:*::: *: :

G.lambia DLLWPEFSVRAFKDCLDEFASRTRRFGGVQ---LSPMTGVYSDTHPHSSTNALSNHD--- 265

E.coli DVLWPDFDEQDFEGALNAFANRERRFGGTE---PGDETA--------------------- 253

M.luteus DEFWPDFNEESLAQCISIYQNRHRRFGGL------------------------------- 249

S.cerevisiae_Srt1 DTLWPNFSFFAMYLMILKWSFFSTIQK-YNEKNHSLFEKI------HESVPSI------- 317

M.acetivorans APFWPEFRKIDLLRSIRVYQARKDEKKQENS----------------YRVSKVINFLGVG 277

S.cerevisiae_Rer2 DCLWPEFGPIRMAWILLKFSFHKSFLNKEYRLEEGDYD-----------------EETNG 275

c.elegans DVLWPEFGYFNLCKAILNYQYYRTTVTKMTSSKVSDDN----------ATSWKMNFSGND 291

Drosophila NVLWPQITFWHFLASILAYQRDRWQLDDFRRAERMQS---CQLAKATDFYS--------- 276

Zebrafish SVLWPEYSFWNLCEAILQFQLSHRSIQ-KARELHREEQVLQQMESDRTCVAEILQHRGNG 287

Cod SVLWPEYSFWNLCDAVIQYQCNHASIM-KARELHQDEQRRQQLEADHACVAELLHHHGNG 287

Pike SVLWPEYTFWNLCEAILQYQLNYRPLQ-KARDQHQEDQAIQQLEADRACVAELIQHCGNG 297

Turbot SVLWPEYSFWNLCEAILQYQLNHKSIQ-KARDLHREHQASLQLEADRACVAEHLQHHGNG 287

eel SVLWPEYSFWNLCEAILQYQLNHKSIQ-KARDLHREHQALQQLEADRDCIAEHLQHHGNG 287

Anole_lizard PVLWPEYSFWNLWEAILQFQMNYNALQ-KARDLYMEERKWQQMETDKACVLEKLKEEGSA 287

chicken SVLWPEYSFWNLCEAILRFQMNYNALQ-KARDSYMEERKQQQMERDQAYVTKKLQQEGFA 287

Duck SVLWPEYSFWNLCEAILRFQMNYSALQ-KARDSYMEERKQQQMERDQAYVTKKLQQEGFA 287

Beaver PILWPEYTFWNLCEAILQFQANHSTLQQKARDLYAEERKRHQLERDQAAVTEQLLQEGLQ 288

Opossum PILWPEYSFWNLCEAILQFQVNHSMLQ-KARDSYMEERKRQQLESDQTAVTEQLLREGCG 287

Mouse PVLWPEYTFWNLCEAILQFQRNHGALQ-KARDMYAEERKRRQLERDQAAVTEQLLREGLQ 287

Rat PVLWPEYTFWNLCEAILQFQMNHSALQ-KARDMYAEERKRHQLERDQAAVTEQLLREGLQ 287

sloth PVLWPEYKFWNLCEAILRFQMNHSMLQ--------------------------------- 255

cat PVLWPEYTFWNLCEALLQYQMNHSMLQQKARDMYAEERKRQQLERDQAAVTEQLLQEGLQ 288

Armadilo PVLWPEYTFWNLCEAILQFQMNHSMLQQKARDMYAEERKRQQLERDQAAVTEQLLQEGLQ 288

Elephant PVLWPEYTFWNLCEAILQFQMNHSALQQKARDMYAEERKRQQLERDQAAVTEQLLQEGLQ 288

Squirrel PVLWPEYTFWNLCEAILQFQMNHSMLQ-KARDTYAEELKRHQLERDQAAVTEQLLREGLQ 287

Dog PVLWPEYTFWNLCEAILQYQMNHSMLQAEGDAKGDKAKVKDEPQRRSARLSAK----PAP 284

Cow PVLWPEYTFWNLCEAILQFQMNHSMLQ-KARDMYAEERKRQQLERDQAAVTEQLLQEGLP 287

Goat PVLWPEYTFWNLCEAILQFQMNHSMLQ-KARDVYAEERKRQQLERDQAAVTEQLLQEGLP 313

DHDDS_human PVLWPEYTFWNLFEAILQFQMNHSVLQ-KARDMYAEERKRQQLERDQATVTEQLLREGLQ 287

Chimpanzee PVLWPEYTFWNLFEAILQFQMNHSVLQQKARDMYAEERKRQQLERDQATVTEQLLREGLQ 288

Rabbit PVLWPEYTFWNLCEAILQFQMNHSMLQ-KARDMYAEERKRHQLERDQAAVTEQLLREGLQ 287

Pig PVLWPEYTFWNLCEAILQFQMNHSMLQ-KARDMYAEERKRQQLERDQAAVTEQLLQEGLQ 287

Alpaca PVLWPEYTFWNLCEAILQFQMNHSVLQ--------------------------------- 255

Horse PVLWPEYTFWNLCEAILQFQMNHSMLQ-KARDMYAEERKRQQLERDQAAVTEQLLQEGLQ 337

:**: : : :

G.lambia ------------------------------------------------------------ 265

E.coli ------------------------------------------------------------ 253

M.luteus ------------------------------------------------------------ 249

S.cerevisiae_Srt1 --FKKKKTAMSLYNFPNPPISVSVTGDE-------------------------------- 343

M.acetivorans KYGEKSEELGQLLPLKKQ----------GV----A------------------------- 298

S.cerevisiae_Rer2 DPIDLKEKKLN------------------------------------------------- 286

c.elegans RDLISVKS---------------------------------------------------- 299

Drosophila ---------------------------ERV-QNFLTTIDEDRR-KLLVRLAAN------- 300

Zebrafish KPMDGQSQQNALLN-------YSSSREERV-RGFLNALQ-HKRDAFFDDLSSQAVVA--- 335

Cod KPADGPRRQEAVAE-------YGLRREARV-KDFLEALR-NKRDSFL------------- 325

Pike NPLDAQRRQEALLQ-------YTASREERV-QDFLCALQ-HKRDSFFTDLTSQAALI--- 345

Turbot KPLDAQRRQEALLH-------YTACREERV-QDFLESLK-HKRDSYFNNLCSEAVLA--- 335

eel KPADAQRRQEALLH-------YAACREERV-QEFLQALK-HKRDSFFNDLCSEAVLA--- 335

Anole_lizard SCADAQRRRTLL-------QELRAEREERV-QGFLEALE-NKRMDFLEKLTVASA----- 333

chicken SHGDSRRRRTLL-------QKCTAMREERI-QGFLQALE-HKRADFFERLCTVSA----- 333

Duck SHGDSQRRRTLL-------QKCTAMREERI-QGFLQALE-HKRADFFERLCTVSA----- 333

Beaver ASEDTQLRRTRL-------HKLLARREERV-QGFLQALE-LKRADWLARLGTASA----- 334

Opossum PSRDSQLRRILL-------HKRSSGREERV-QGFLQALE-RKREEWLARLGTASS----- 333

Mouse ASGDAQLRRTRL-------HKLSTKREERV-QGFLKALE-LKRANWLALWGTASA----- 333

Rat ANGDAQLRRTRL-------HKLSAKREERV-RGFLQALN-LKRADWLALWGTASA----- 333

sloth ------------------------------------------------------------ 255

cat ASGDAQLRRTRL-------HKLSARREERV-QGFLQALE-LKRADWLARLGTASA----- 334

Armadilo ASGDAQLRRTCL-------HKLSARREERV-QGFLQALE-FKRADWLARLGTASA----- 334

Elephant ASGDAQLRRTFL-------HKLSARREERV-QGFLQALE-LKRADWLARLGTASA----- 334

Squirrel ASGDAQLRRTRL-------HKLSARREERV-QGFLQALK-LKRANWLARLGTASA----- 333

Dog PKPEPKP---KKAP---------AKKGEKVPKGKKGKADAGKDGNNPAENGDAKTDQAQK 332

Cow ASGDPQLRRTRL-------HKLSARREERV-QGFLQALE-LKRADWLAHLGTAST----- 333

Goat ASGDPQLRRTRL-------HKLSARREERV-QGFLQALE-LKRADWLAHLGTAST----- 359

DHDDS_human ASGDAQLRRTRL-------HKLSARREERV-QGFLQALE-LKRADWLARLGTASA----- 333

Chimpanzee ASGDAHLRRTRL-------HKLSARREERV-QGFLQALE-LKRADWLARLGTASA----- 334

Rabbit VWRAGRHSALAACPFRLCHHSLCAP----G-RTFLATAH--PSANNCREEGTPSLPEP-- 338

Pig ASGDTQLRRTRL-------HKLLARREERV-QVFLQALE-LKRADWLARLGTASA----- 333

Alpaca ------------------------------------------------------------ 255

Horse ASGDAQLRWTRL-------HKLSARREERV-QGFLQALE-LKRADWLARLGTASA----- 383

G.lambia -------- 265

E.coli -------- 253

M.luteus -------- 249

S.cerevisiae_Srt1 -------- 343

M.acetivorans -------- 298

S.cerevisiae_Rer2 -------- 286

c.elegans -------- 299

Drosophila -------- 300

Zebrafish -------- 335

Cod -------- 325

Pike -------- 345

Turbot -------- 335

eel -------- 335

Anole_lizard -------- 333

chicken -------- 333

Duck -------- 333

Beaver -------- 334

Opossum -------- 333

Mouse -------- 333

Rat -------- 333

sloth -------- 255

cat -------- 334

Armadilo -------- 334

Elephant -------- 334

Squirrel -------- 333

Dog AEGAGDAK 340

Cow -------- 333

Goat -------- 359

DHDDS_human -------- 333

Chimpanzee -------- 334

Rabbit -------- 338

Pig -------- 333

Alpaca -------- 255

Horse -------- 383
