# Supplementary material for: Structural basis of heterotetrameric assembly and disease mutations in the human cis-prenyltransferase complex
Source: Nat Commun. 2020 Oct 19;11:5273. doi: 10.1038/s41467-020-18970-z (PMC7573591; doi:10.1038/s41467-020-18970-z)
Supplement: Supplementary file 4 — Reporting Summary [file 41467_2020_18970_MOESM4_ESM.pdf]

nature research

Corresponding author(s): Yoni Haitin, Moshe Giladi

Last updated by author(s): Sep 19, 2020

Reporting Summary

Nature Research wishes to improve the reproducibility of the work that we publish. This form provides structure for consistency and transparency in reporting. For further information on Nature Research policies, see our Editorial Policies and the Editorial Policy Checklist.

Statistics

For all statistical analyses, confirm that the following items are present in the figure legend, table legend, main text, or Methods section.

n/a	Confirmed	
☐	☒	The exact sample size (*n*) for each experimental group/condition, given as a discrete number and unit of measurement
☐	☒	A statement on whether measurements were taken from distinct samples or whether the same sample was measured repeatedly
☐	☒	The statistical test(s) used AND whether they are one- or two-sided<br>*Only common tests should be described solely by name; describe more complex techniques in the Methods section.*
☒	☐	A description of all covariates tested
☐	☒	A description of any assumptions or corrections, such as tests of normality and adjustment for multiple comparisons
☐	☒	A full description of the statistical parameters including central tendency (e.g. means) or other basic estimates (e.g. regression coefficient) AND variation (e.g. standard deviation) or associated estimates of uncertainty (e.g. confidence intervals)
☐	☒	For null hypothesis testing, the test statistic (e.g. *F*, *t*, *r*) with confidence intervals, effect sizes, degrees of freedom and *P* value noted<br>*Give P values as exact values whenever suitable.*
☒	☐	For Bayesian analysis, information on the choice of priors and Markov chain Monte Carlo settings
☒	☐	For hierarchical and complex designs, identification of the appropriate level for tests and full reporting of outcomes
☒	☐	Estimates of effect sizes (e.g. Cohen's *d*, Pearson's *r*), indicating how they were calculated

Our web collection on statistics for biologists contains articles on many of the points above.

Software and code

Policy information about availability of computer code

Data collection	X-ray crystallography: Generic Data Acquisition 9.0 (Diamond light source, UK).
Data analysis	Native ESI: MassLynx 4.1.<br>Enzyme kinetics: Origin 7.0, Graphpad Prism 8.<br>X-ray crystallography: XDS, coot 0.8, Phenix 1.14-3260, Mr Bump 2.0.5, CCP4 online 1.1.1, UCSF chimera 1.14, MolProbity 4.02b-467.<br>Molecular dynamics: Schrodinger's Maestro 11.2, GROMACS 2018.2, VMD 1.9.3.<br>Sequence alignment: ClustalW2.

For manuscripts utilizing custom algorithms or software that are central to the research but not yet described in published literature, software must be made available to editors and reviewers. We strongly encourage code deposition in a community repository (e.g. GitHub). See the Nature Research guidelines for submitting code & software for further information.

Data

Policy information about availability of data

All manuscripts must include a data availability statement. This statement should provide the following information, where applicable:

- Accession codes, unique identifiers, or web links for publicly available datasets
- A list of figures that have associated raw data
- A description of any restrictions on data availability

Atomic coordinates and structure factors for the structure of xcis-PT in complex with Mg2+ and FPP have been deposited in the Protein Data Bank with accession number 6Z1N [https://doi.org/10.2210/pdb6Z1N/pdb].

Field-specific reporting

Please select the one below that is the best fit for your research. If you are not sure, read the appropriate sections before making your selection.

☒ Life sciences ☐ Behavioural & social sciences ☐ Ecological, evolutionary & environmental sciences

For a reference copy of the document with all sections, see nature.com/documents/nr-reporting-summary-flat.pdf

Life sciences study design

All studies must disclose on these points even when the disclosure is negative.

Sample size	No statistical methods were used to predetermine sample sizes. Required experimental sample sizes were chosen according to common practice in enzymology (at least three independent experiments, as indicated in the figure legends). Statistical analysis was limited to determine mean ± SD.
Data exclusions	Data were not excluded.
Replication	We have purified each protein at least two times and ran SEC-MALS for each protein batch to validate the oligomeric state. All attempts at replication were successful.<br>Cross-linking and mass-spectrometry experiments were repeated three and two times, respectively. All attempts at replication were successful.<br>Enzyme kinetics experiments with the purified enzyme were repeated in order to ensure reproducibility as follows: In titration experiments, each data points was repeated 3 times. In the mutant analysis, each variant was measured 5-7 times. Furthermore, we have sampled several mutants from different batches to ensure that the mutational effect is reproducible compared to the WT. All attempts at replication were successful.<br>Molecular dynamics simulations were performed in triplicates to ensure reproducibility. All attempts at replication were successful.
Randomization	Randomization was not performed in this study as samples were predefined and did not require allocation into different experimental groups. The groups were prepared independently and compared under controlled conditions.
Blinding	Blinding was not performed in this study as the results are quantitative in nature and do not require subjective interpretation.

Reporting for specific materials, systems and methods

We require information from authors about some types of materials, experimental systems and methods used in many studies. Here, indicate whether each material, system or method listed is relevant to your study. If you are not sure if a list item applies to your research, read the appropriate section before selecting a response.

Materials & experimental systems

n/a	Involved in the study	
☒	☐	Antibodies
☒	☐	Eukaryotic cell lines
☒	☐	Palaeontology and archaeology
☒	☐	Animals and other organisms
☒	☐	Human research participants
☒	☐	Clinical data
☒	☐	Dual use research of concern

Methods

n/a	Involved in the study	
☒	☐	ChIP-seq
☒	☐	Flow cytometry
☒	☐	MRI-based neuroimaging
