# Supplementary material for: Structural basis of heterotetrameric assembly and disease mutations in the human cis-prenyltransferase complex
Source: Nat Commun. 2020 Oct 19;11:5273. doi: 10.1038/s41467-020-18970-z (PMC7573591; doi:10.1038/s41467-020-18970-z)
Supplement: Supplementary file 2 — Peer Review File [file 41467_2020_18970_MOESM2_ESM.pdf]

REVIEWER COMMENTS

Reviewer #1 (Remarks to the Author):

The manuscript titled "Structural basis of heterotetrameric assembly and disease mutations in the human cisprenyltransferase complex" by Bar-El et al, is a timely study elucidating the structure of the catalytic domain of the human cis-isoprenyltransferase complex. Mutations in the genes coding for DHDDS and NgBR, are causative of Retinitis Pigmentosa (RP59) and early onset encephalopathy. The structural elucidation, specifically that of the catalytic domain, further attempts to explain the molecular underpinning of RP59 as pertains to K42E point mutation.

1. The hCIT complex is involved in synthesis of dolichol, the obligate lipid carrier for N-glycosylation. In the introduction, the authors refer to causative mutations (K42E, and severe mutation) of DHDDS leading to RP59, as CDG-causing. However, no glycosylation or mannose incorporation defects has been shown in K42E patient samples, or even in the severe case of DHDDS-associated RP59 (References 11,12,14). In fact, recent studies modeling RP59 in mice (through K42E knockin mouse model, and conditional deletion of DHDDS in photoreceptors and RPE) [See pertinent PMIDs: 32272552, 32526701, 32245241] shows retinal degeneration, independent of N-glycosylation defects, suggesting a complex cell biological mechanism underlying RP59. Rhodopsin and LAMP2 glycosylation was not perturbed in the retina of both the knockin and knockout mouse models. However, unlike the K42E mutation (or the severe mutation) in DHDDS, the R290H mutation in NgBR mutation clearly exhibits N-glycosylation defects (See LAMP2 glycosylation defects in patient fibroblasts in Ref #15). Therefore the disease mechanism of DHDDS-associated RP59 may be different compared to NgBR R290H mutation-associated visual deficits. The introduction should be more carefully laid out and reflect the above, recent understanding of RP59, since no strong evidence for CDG exist to suggest the same.

2. The study clearly validates and demonstrates the formation of heterotetramer, and the residues involved in binding to Mg2+, FPP and IPP binding. Previous studies have similarly determined FPP and IPP binding sites in E.coli UPPS (Ref # 5). The manuscript would greatly benefit by a supplementary figure demonstrating protein sequence alignment across species (E.coli, M.musculus, H.sapiens, along with the demarcation of alpha helices) (See supplementary Figs PMID: 32526701) to demonstrate critical binding pockets.

3. The study suggests that the K42E mutation decreases the catalytic activity of DHDDS.

However, the biochemical manifestation of the K42E and T206A mutation, as observed in patient samples (See PMID: 24078709, is decrease in the dolichol chain length by one isoprene unit. The MSD simulations in the study does not fully explain these observations. Decrease in dolichol chain length by 1 IPP unit would suggest specific deficiency of a IPP-IPP condensation reaction, instead of a decreased overall catalytic activity of DHDDS complex. The current conclusions do not fully explain the increased D18/D19 ratio in patients with K42E mutation. The authors should also replicate similar MSD simulations in T206A mutation of DHDDS, as well as the severe mutations published in Ref #14.

Reviewer #2 (Remarks to the Author):

The manuscript entitled Structural basis of heterotetrameric assembly and disease mutations in the human cis-prenyltransferase complex describes biochemical and structural properties of human DHDDS. The authors can hence inspect the effects of inherent disease-related DHDDS mutations basing on structure information. This study is a milestone for isoprenoid synthase research because this is the first structure of the asymmetric cis-prenyltransferase. Furthermore, these knowledge should greatly advance our understanding into dolichol biosynthesis, as well as the development of disease therapy. The experiments are conducted in sound techniques and presented properly. A few comments should be addressed prior to be accepted for publication.

Comments
1. The authors referenced several review articles for cis-PTs background but a more update review by Chen et al. which summarizes structures and catalytic mechanisms of cis-prenyltransferases is missing (https://dx.doi.org/10.1021/acscatal.0c00283). This article should be cited for more comprehensive understanding of these enzymes.
2. Figure S1. This figure was brought up before it is elaborated in the result section so it is hard to understand symbols. Please supplement the necessary information in the figure legend so that readers can understand easier.
3. Page 4, 2nd paragraph, 1st sentence. In addition to prokaryotic cis-PTs, many plant and fungal cis-PTs are also extensively studies. Please refer to Chen’s review (comment 1).
4. In addition to the conventional cis-PTs, the crystal structure of NgBR homolog in yeast (Nus1) has been recently reported. The authors should describe Nus1 structure to provide a more comprehensive information in the introduction section.
5. Result section, P. 6, section 2, 2nd paragraph, line 6. Superposition of DHDDS catalytic domain and other cis-PT homolog (say UPPS or DPPS) should be provided in addition to their

RMSD values.

6. The Nus1 structure paper (#19) should be referenced in the 1st paragraph on page 7, and a detailed structural comparison of NgBR and Nus1 is strongly recommended. Nus1 also lacks catalytic features as NgBR does and it should be informative to show the structural comparison of these two proteins.

7. The homodimer of heterodimer configuration is very interesting. Could the author try to depict the membrane localization pattern of the whole protein complex? One may find this information very useful in learning how DHDDS practices catalysis in its cellular destination.

8. P. 9, the hydrophobic tunnel between helix alpha 2 and alpha 3 deserves more discussions. For instance, what is the corresponding residue of the N-terminal W3 in other homologs?

9. P. 11, 2nd paragraph, the descriptions concerning C148 should be included in the Result section, such that the overall picture of the substrate/product-binding pocket can be revealed.

Reviewer #3 (Remarks to the Author):

This manuscript reports the crystal structure of a long-chain *cis*-prenyltransferase from human. Although the first crystal structure of *cis*-prenyltransferase was determined around 20 years ago, long-chain *cis*-prenyltransferase is different from the determined *cis*-prenyltransferases in some points. Especially, all determined *cis*-prenyltransferases are homodimeric, whereas human long-chain *cis*-prenyltransferase is a heteromeric enzyme. This manuscript clearly describes the catalytic site environment constructed by the two subunits. In addition, the structure shed lights onto some congenital diseases related to long-chain *cis*-prenyltransferase. Thus, the manuscript is expected to have high impact to the science community.

However, the present manuscript lacks several essential experimental evidences. Thus, this paper is recommended to invite the revision with the following comments.

Major points

1. Crystal structure

The coordinates of the crystal structure should be considered just as the “interpretation” of the experimental data. Non-model-biased electron density map is required to judge the validity of the coordinates. However, the authors only show $2F_o$-F_c map around the ligand FPP in Figure 4B. $2F_o$-F_c map is NOT suitable to show the existence of the ligand, because the map is biased by the model. Instead, F_o-F_c omit electron density map must be used.

Sometime, F_o-F_c (or mF_o-dF_c) omit electron density map is very noisy. In such case, polder map (https://www.phenix-online.org/documentation/reference/polder.html) may improve the map quality. If the ligands (including Mg^{2+} and special water etc.) are not clearly seen in the polder map, the ligands etc. should not be included in the coordinates.

The authors should show non-biased electron density map for ligands (FPP, phosphate and Mg^{2+}), and several interesting residues such as ligand-interacting residues, C-terminal residues around NgBR.

2. SDS-PAGE

The authors may want to represent the purity of their sample by the SDS-PAGE in Figure 2B. For this purpose, the top and the bottom parts of the lane should not be omitted.

In Figure 2C, molecular weight markers are essential to interpret the PAGE. The authors must show the marker lane in the panel. If there are no space to show the marker lane, the full PAGE

image may be shown in the supplementary figure.

3. Biological assembly

The authors discuss the oligomeric state of DHDDS and NgBR in P7. The authors are recommended to analyze their structure by PDBePISA (https://www.ebi.ac.uk/pdbe/pisa/). The service analyzes whether the interface of the components in the crystal is biological assembly or not.

4. No substrate binding site in NgBR

The authors refer Figure 4C to show no pocket in NgBR. However, I am wondering that a solvent accessible pocket might be seen from a different angle.

Probably, some part of the structure in figure 4C might be superposed on the coordination used to draw figure 4A, and the two figures were drawn from the same angle. Correct? Anyway, please describe how the figure is drawn in detail.

Regarding figure 4D, the authors may want to show that several strands and helices of NgBR occupy the substrate binding cavity on DHDDS (text P9 L29-33). If correct, please clearly represent the substrate binding site in DHDDS in Figure 4D.

5. MD simulation (P.10 L8-24)

The authors describe the distance distributions in Figures 5D and 5E in order to show the interactions between 42 and 234, as well as between 42 and 38. However, it sounds nonsense to show the distance distribution between the positively charged heads of K42 and R38, and between the negatively charged carboxylates of E42 and E234. It is not surprising the distance distribution of K42E is wider than that for WT in Figure 5D, because the former does not form attractive interaction. I obtained a similar impression from Figure 5E.

How are the distance distributions between the corresponding Cα atoms? Showing the distance distributions for Cα atoms together with those for the charged group may strengthen the discussion.

Also, how are the B-factor of the amino head of K42 and carboxylate group of E234? They should be reasonably low comparing to those for the surrounding atoms, since the two residues are suggested to form a salt bridge.

6. Labels in the figures

Some texts are too small to read. For example, the labels DHDDS and NgBr in Figure 1A are too small. At least 6 points are recommended except for the super/sub-scripts. Please update ALL figures.

It is hard to see the color for fonts with black rims, especially the small labels such as Figure 2. Please consider using the dark color with the same hue, and remove the black rim.

Minor points

7. Figure 1A

Isoprene unit colored in red should be *cis*-isoprene. Please correct.

8. P.8. L.20

The authors refer Figure 3D. Is this correct? If yes, what is βB-α3 linker? By the caption for figure 3D, this figure may represent S2 site, but the sentence describes S1 site. I am confused.

9. P.11 L3.3

Please show where the helix-turn-helix motif following α7 is in Figure 2.

0. P.12 L.17

Normally, a lysine does not attractively interact with arginines. So the sentence “K293* interacts with...” sounds strange. For the reader’s convenience, please specify the carboxylate of C-terminal at K293* interact with arginines.

1. Units

- A space is required before the degree unit “°C”. For example, “16°C” in P.14, L.10 and L.11.
- A space is required before the voltage unit “V” or “kV”. For example, “40V”, “20V” and “1.8kV” in P.14, L.31-32.
- A space is required before the flow-rate unit “ml/min”. For example, “4ml/min” in P.14, L.31.
- Please unify either “h” or “hour” throughout the manuscript. For example, “16-20 h” in P.14, L.12 and “1 hour” in P.15, L.18”.
- Please replace “°” into a space at “100°K” in P.15, L.14.
- Insert a space in “5Å” in P. 25, L.13.

2. Abbreviations

All abbreviation should be spelled out at their first appearance. For example, “HEPES”, “TCEP” in P.14 L17 and L18.

3. P.15, L.3.

Km may be K_M

14. P.15 L.13.
What is NaP?

15. P16. L.4
Please remove underline between the "(WT and mutant)" and "The resulting".

16. Table 1
- Please show the resolution range for the full dataset (not only in the parenthesis).
- "R" of "R_{meas}", "R_{work}", "R_{free}", "CC" of "$CC_{1/2}$", "I" of Mean $I/\sigma(I)$ should be italicized.
- Please add the unit (Å) for the "Resolution range" line at the "Refinement statistics".
- Please add the units (Å, °) for the "RMSD (bond lengths/angles)" lines.
- Please describe what programs/servers are used to perform the ramachandran analysis with the proper citations. Also, please provide the rate of favorite/acceptable residues by the ramachandran analysis.

17. Supplementary Figure S3
20 mM FPP and 100 mM IPP may be typo. mM may be μM.

18. Supplementary Figure S4
What is the difference between the panels A and B? I mean, why do the authors separate the 8 SEC-MALS analyses into 2 parts? Is there any difference between the red (S4A) and green (S4B) curves? Also, I am wondering why the molecular weight is shown in blue in Figure S3A. If there are no experimental difference, drawn in the same color is preferred.

Reviewer #4 (Remarks to the Author):

In this manuscript, high-resolution crystal structure has been determined for the human cis-prenyltransferase (hcis-PT), which is an essential enzyme for protein N-clycosylation and implicated in human diseases including autosomal recessive retinitis pigments (arRP), epilepsy, neurological impairments and hearing deficit. Disease mutations of hcis-PT around the active site have been investigated through combined experiments and molecular dynamics (MD) simulations, which provide intriguing mechanistic insights into the enzyme dysfunction in arRP. Overall, the manuscript is well written. The results are supported by the presented data. For the MD simulations, the manuscript can be still improved with the following major suggestions:

1. Three 100 ns MD simulations have been performed on both the WT and K42E mutant of hcis-PT. But why are results shown for only one of the three simulations in Fig. 5 and Fig. S8? All the simulations should be analyzed carefully. The results need to be presented for individual simulations and compared to evaluate the simulation convergence, especially given that 100 ns seems pretty short with state-the-of-art MD simulations.

2. Related to the above point, in Fig. S8 it may help to plot the averages and standard deviations (error bars) of the residue RMSFs that can be calculated from three individual simulations of each system.

3. In Fig. 5D, the distance was "measured between the carboxylate oxygens of E234 and the side-chain nitrogen (Nz) of K42 (light blue) or the carboxylate oxygens of E42 (light orange)." This may lead to multiple atom distances given the description. Do the authors calculate the minimum atom distance of their measurements between two interested residues? This is not clear and needs clarification, similarly for the distance plots in Fig. 5E. Alternatively, the authors can probably use charge centers of the basic and acidic residue side chains to avoid such confusion and fluctuations of the distance plots.

4. In addition to the RMSD plot in Fig. 5G, it would help to compare the simulation final structure of the K42E mutant with the WT, especially for the enzyme active site

Sackler Faculty of Medicine
Tel Aviv University

Laboratory of structural physiology | Dept. of Physiology and Pharmacology | Tel Aviv, Israel 6997801 | office 972-3-6409261 | fax 972-3-6409894

Reviewer #1:

The manuscript titled "Structural basis of heterotetrameric assembly and disease mutations in the human *cis*-prenyltransferase complex" by Bar-El et al, is a timely study elucidating the structure of the catalytic domain of the human *cis*-isoprenyltransferase complex. Mutations in the genes coding for DHDDS and NgBR, are causative of Retinitis Pigmentosa (RP59) and early onset encephalopathy. The structural elucidation, specifically that of the catalytic domain, further attempts to explain the molecular underpinning of RP59 as pertains to K42E point mutation.

Response: We thank reviewer #1 for the insightful comments and suggestions.

1. The hCIT complex is involved in synthesis of dolichol, the obligate lipid carrier for N-glycosylation. In the introduction, the authors refer to causative mutations (K42E, and severe mutation) of DHDDS leading to RP59, as CDG-causing. However, no glycosylation or mannose incorporation defects has been shown in K42E patient samples, or even in the severe case of DHDDS-associated RP59 (References 11,12,14). In fact, recent studies modeling RP59 in mice (through K42E knockin mouse model, and conditional deletion of DHDDS in photoreceptors and RPE) [See pertinent PMIDs: 32272552, 32526701, 32245241] shows retinal degeneration, independent of N-glycosylation defects, suggesting a complex cell biological mechanism underlying RP59. Rhodopsin and LAMP2 glycosylation was not perturbed in the retina of both the knockin and knockout mouse models. However, unlike the K42E mutation (or the severe mutation) in DHDDS, the R290H mutation in NgBR mutation clearly exhibits N-glycosylation defects (See LAMP2 glycosylation defects in patient fibroblasts in Ref #15). Therefore the disease mechanism of DHDDS-associated RP59 may be different compared to NgBR R290H mutation-associated visual deficits. The introduction should be more carefully laid out and reflect the above, recent understanding of RP59, since no strong evidence for CDG exist to suggest the same.

Response: We thank the reviewer for raising this important point. We agree that although we did not explicitly state that altered glycosylation is the common mechanism leading to the clinical phenotypes caused by h*cis*-PT mutations, it can be easily inferred by the reader. Thus, we clarified this point in the revised manuscript, at the end of the 3rd paragraph of the introduction:

"*Intriguingly, the different pathogenic mutations in hcis-PT seem to have diverse effects on cellular glycosylation. For example, while the missense mutation in the RxG motif of NgBR led to reduced glycosylation in patients fibroblasts* [15]*, the arRP mutation in DHDDS does not seem to have any significant effect on glycosylation in a knock-in mouse model* [17,18]*. Thus, the interplay between the genotype and cellular phenotype may be more complex than originally thought and awaits further exploration."*

2. The study clearly validates and demonstrates the formation of heterotetramer, and the residues involved in binding to Mg^{2+}, FPP and IPP binding. Previous studies have similarly determined FPP and IPP binding sites in *E. coli* UPPS (Ref # 5). The manuscript would greatly benefit by a supplementary figure demonstrating protein sequence alignment across species (E.coli, M.musculus, H.sapiens, along with the demarcation of alpha helices) (See supplementary Figs PMID: 32526701) to demonstrate critical binding pockets.

Response: Thank you for this suggestion. While these primary-sequence alignments are insightful, the structure provided in this manuscript enables us to enhance the reader's grasp of the conserved domains even

more through the use of the ConSurf server (https://consurf.tau.ac.il). In line with your suggestion, we have added the new Figure S6, with the structure of DHDDS color-coded according to the conservation score of each residue, along with the sequence alignment used for this analysis. This analysis is now referenced throughout the manuscript.

3. The study suggests that the K42E mutation decreases the catalytic activity of DHDDS. However, the biochemical manifestation of the K42E and T206A mutation, as observed in patient samples (See PMID: 24078709, is decrease in the dolichol chain length by one isoprene unit. The MSD simulations in the study does not fully explain these observations. Decrease in dolichol chain length by 1 IPP unit would suggest specific deficiency of a IPP-IPP condensation reaction, instead of a decreased overall catalytic activity of DHDDS complex. The current conclusions do not fully explain the increased D18/D19 ratio in patients with K42E mutation. The authors should also replicate similar MSD simulations in T206A mutation of DHDDS, as well as the severe mutations published in Ref #14.

Response: As the reviewer indicated, the dolichol profiling analysis of patients samples revealed that serum and plasma from both healthy and affected subjects contained D17, D18, D19 and D20. However, the relative abundance of each dolichol specie is altered, with increased abundance of the shorter dolichol species in RP 59 patients. Although this altered distribution results in an altered D18/D19 ratio, in our opinion, it does not reflect a specific deficiency of a *single* IPP condensation reaction. Rather, the altered distribution can reflect weaker binding of the product at the active site, resulting in a higher propensity for premature release, as originally suggested by Ren et al. Moreover, as the manuscript by Ren et al. references relative abundances only, no quantitative measure of the overall dolichols species amounts was made. Thus, the observation that the K42E mutation decreases the catalytic activity at the protein level does not contradict their results.

Our MD simulations reveals that the K42E mutation results in active site destabilization, including the C-terminus of NgBR, as reflected by the overall increase in RMSD. We have addressed this issue in the discussion and suggested that the globally enhanced dynamics of the active site may result in weakening of long-chain products binding, leading to premature product release, explaining the shift of the dolichols population in addition to the reduction in catalytic activity, as described by Ren et al.

Finally, the T206A mutant allele was described only in one patient, which was also heterozygous for the K42E allele. Hence, we did not address this mutation in our manuscript, since its pathogenic role requires further validation. Importantly, per the reviewer's request, we performed MD simulations of the complex harboring DHDDS-T206A, revealing that similar to DHDDS-K42E, the active site exhibits globally enhanced dynamics (see attached figure). However, as the role of this mutant in human disease remains to be determined, we feel that discussion of this mutant is beyond the scope of this manuscript. The severe mutations published in Ref #14 result in early termination due to a splice site mutation and a nonsense mutation, and thus, they cannot be modelled.

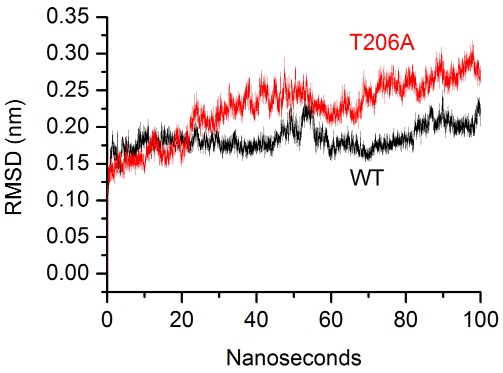

Reviewer #2:

The manuscript entitled Structural basis of heterotetrameric assembly and disease mutations in the human cis-prenyltransferase complex describes biochemical and structural properties of human DHDDS. The authors can hence inspect the effects of inherent disease-related DHDDS mutations basing on structure information. This study is a milestone for isoprenoid synthase research because this is the first structure of the asymmetric cis-prenyltransferase. Furthermore, these knowledge should greatly advance our understanding into dolichol biosynthesis, as well as the development of disease therapy. The experiments are conducted in sound techniques and presented properly. A few comments should be addressed prior to be accepted for publication.

Response: We thank reviewer #2 for this overview of the manuscript and the useful comments and suggestions.

Comments

1. The authors referenced several review articles for cis-PTs background but a more update review by Chen et al. which summarizes structures and catalytic mechanisms of cis-prenyltransferases is missing (https://dx.doi.org/10.1021/acscatal.0c00283). This article should be cited for more comprehensive understanding of these enzymes.

Response: Thank you for suggesting this comprehensive and updated review. We now cite it throughout the revised manuscript (Ref #4).

2. Figure S1. This figure was brought up before it is elaborated in the result section so it is hard to understand symbols. Please supplement the necessary information in the figure legend so that readers can understand easier.

Response: Thank you for this suggestion. We have expanded the figure legend to enhance understanding:

"The secondary structures of DHDDS ***(a)*** *and sNgBR* ***(b)*** *are indicated above the sequence. The N-terminal domain of DHDDS and NgBR include a single α-helix, αN. The cis-prenyltransferase homology domain of DHDDS includes 7 α-helices (α1–7) and 6 β-strands (βA-F), while the pseudo cis-prenyltransferase homology domain of NgBR includes 6 α-helices (α1–6) and 5 β-strands (βA-E). Finally, the C-terminal domain of DHDDS includes two α-helices, designated αC1 and αC2, while the C-terminus of NgBR includes the conserved RxG motif."*

3. Page 4, 2nd paragraph, 1st sentence. In addition to prokaryotic cis-PTs, many plant and fungal cis-PTs are also extensively studies. Please refer to Chen's review (comment 1).

Response: Thank you, corrected and cited:

"Despite an immense body of work focusing on the biochemical and structural properties of cis-prenyltransferases, our mechanistic understanding of these enzymes arises mainly from investigations of homodimeric prokaryotic, plant and fungal orthologs [2,4]."

4. In addition to the conventional cis-PTs, the crystal structure of NgBR homolog in yeast (Nus1) has been recently

reported. The authors should describe Nus1 structure to provide a more comprehensive information in the introduction section.

Response: Thank you for this suggestion. The structure of Nus1 is now referenced in the 4th paragraph of the introduction:

"Recently, the structure of Nus1, the yeast homolog of NgBR, was determined. The structure, devoid of the N-terminal transmembrane domain, revealed that similarly to DHDDS, Nus1 can form homodimers when expressed alone[22]. However, Nus1 does not contain the canonical RxG motif. Instead, it contains a smaller and neutral asparagine residue[22]. Thus, the quest for elucidating the functional and structural roles of NgBR activity in the context of the hcis-PT complex is still ongoing and the underlying mechanism remains to be determined."

In addition, we compare the structures in the last paragraph of the structure overview section and in a new supplementary figure:

"Moreover, NgBR and its yeast homology, Nus1, do not share high structural similarity (RMSD = 1.33Å), with the $\alpha3$ of NgBR occupying the position of the anti-parallel βC-βC' in Nus1 (Figure S5)."

5. Result section, P. 6, section 2, 2nd paragraph, line 6. Superposition of DHDDS catalytic domain and other cis-PT homolog (say UPPS or DPPS) should be provided in addition to their RMSD values.

Response: We added Figure S5 in line with your suggestion:

"This domain shares high homology with undecaprenyl diphosphate synthase (UPPS), a bacterial medium-chain cis-prenyltransferase homolog, with RMSD = 0.76Å [6] (Figures S5, S6)."

6. The Nus1 structure paper (#19) should be referenced in the 1st paragraph on page 7, and a detailed structural comparison of NgBR and Nus1 is strongly recommended. Nus1 also lacks catalytic features as NgBR does and it should be informative to show the structural comparison of these two proteins.

Response: We now compare between NgBR and Nus1, as described in the response to comments 4 and 5.

"Moreover, NgBR and its yeast homology, Nus1, do not share high structural similarity (RMSD = 1.33Å), with the $\alpha3$ of NgBR occupying the position of the anti-parallel βC-βC' in Nus1 (Figure S5)."

7. The homodimer of heterodimer configuration is very interesting. Could the author try to depict the membrane localization pattern of the whole protein complex? One may find this information very useful in learning how DHDDS practices catalysis in its cellular destination.

Response: We completely agree with the reviewer that this is intriguing and may be useful in understanding of the catalytic activity in the cellular context. However, the structure of the N-terminus of NgBR is unknown and even the basic topology of the transmembrane helices is debated. Thus, we feel that currently the available data are insufficient to reliably make this important prediction.

8. P. 9, the hydrophobic tunnel between helix alpha 2 and alpha 3 deserves more discussions. For instance, what is the corresponding residue of the N-terminal W3 in other homologs?

Laboratory of structural physiology | Dept. of Physiology and Pharmacology | Tel Aviv, Israel 6997801 | office 972-3-6409261 | fax 972-3-6409894

Response: We performed a conservation analysis of DHDDS, now presented in Figure S6. This analysis indicate that W3 is largely conserved among the examined species, as now indicated in the manuscript.

"Moreover, the conserved N-terminal W3 (Figure S6) interacts with F55, F101 and V152, thereby occluding the outlet of the hydrophobic tunnel of the active site."

In addition, in line with comment 9 (see below), we now also discuss the hydrophobic tunnel outlet in this section.

9. P. 11, 2nd paragraph, the descriptions concerning C148 should be included in the Result section, such that the overall picture of the substrate/product-binding pocket can be revealed.

Response: According to your suggestion, we moved the descriptions regarding C148 to the results section and left only the proposed role of C148 in the discussion:

"*In addition to the active site diameter, the composition of its terminal region also plays a key role in determination of product length. In UPPS, L137, localized to the N-terminus of βD, was shown to be vital for determining chain length, with the L137A mutant increasing product length from* C_{55} *to* C_{75}[24]*. Our structure reveals that the corresponding position in DHDDS is replaced by C148, a hydrophilic and less bulky residue. Thus, C148 cannot occlude the hydrophobic tunnel outlet as efficiently as L137, similar to the L137A mutant, possibly contributing to long-chain products formation."*

Reviewer #3:

This manuscript reports the crystal structure of a long-chain *cis*-prenyltransferase from human. Although the first crystal structure of *cis*-prenyltransferase was determined around 20 years ago, long-chain *cis*-prenyltransferase is different from the determined *cis*-prenyltransferases in some points. Especially, all determined *cis*-prenyltransferases are homodimeric, whereas human long- chain *cis*-prenyltransferase is a heteromeric enzyme. This manuscript clearly describes the catalytic site environment constructed by the two subunits. In addition, the structure shed lights onto some congenital diseases related to long-chain *cis*-prenyltransferase. Thus, the manuscript is expected to have high impact to the science community.

Response: We thank the reviewer for the thorough assessment and insightful comments.

However, the present manuscript lacks several essential experimental evidences. Thus, this paper is recommended to invite the revision with the following comments.

Major points

1. Crystal structure

The coordinates of the crystal structure should be considered just as the “interpretation” of the experimental data. Non-model-biased electron density map is required to judge the validity of the coordinates. However, the authors only show $2F_O$-F_C map around the ligand FPP in Figure 4B. $2F_O$-F_C map is NOT suitable to show the existence of the ligand, because the map is biased by the model. Instead, F_O-F_C omit electron density map must be used.

Sometime, F_O-F_C (or mF_O-dF_C) omit electron density map is very noisy. In such case, polder map (https://www.phenix-online.org/documentation/reference/polder.html) may improve the map quality. If the ligands (including Mg^{2+} and special water etc.) are not clearly seen in the polder map, the ligands etc. should not be included in the coordinates.

The authors should show non-biased electron density map for ligands (FPP, phosphate and Mg^{2+}), and several interesting residues such as ligand-interacting residues, C-terminal residues around NgBR.

Response: Thank you for this suggestion. In line with your comment, we have generated omit maps of (1) the ligands (FPP, phosphate, Mg^{2+} and its coordinating water molecules), (2) the C-terminus of NgBR (residues 289-293, including the RxG motif) and (3) the ligand coordinating residues in DHDDS (D34, R37, R38, R85, R205, R211, S213). The densities are clearly visible in all the omit maps as shown in the new Figure S4.

2. SDS-PAGE

The authors may want to represent the purity of their sample by the SDS-PAGE in Figure 2B. For this purpose, the top and the bottom parts of the lane should not be omitted.

In Figure 2C, molecular weight markers are essential to interpret the PAGE. The authors must show the marker lane in the panel. If there are no space to show the marker lane, the full PAGE image may be shown in the supplementary figure.

Response: We agree with the reviewer that showing the full lane and marker is needed in this case. Thus, the SDS-PAGE including the marker lane are now shown in Figures 1b,c:

*"**(b)** SEC-MALS analysis of the purified shcis-PT. The black and red curves indicate UV absorption and molecular weight, respectively. Inset: SDS-PAGE analysis of the purified complex. Left lane: molecular weight marker, right lane: purified shcis-PT. Molecular weights are indicated. **(c)** Native ESI-MS spectrum obtained using low activation conditions. The main distribution corresponds to the heterotetramer (charge states 21-26). Inset: SDS-PAGE analysis of the complex following glutaraldehyde cross-linking. Left lane: molecular weight marker, middle lane: purified shcis-PT without glutaraldehyde, right lane: purified shcis-PT with glutaraldehyde. Molecular weights are indicated."*

3. Biological assembly
The authors discuss the oligomeric state of DHDDS and NgBR in P7. The authors are recommended to analyze their structure by PDBePISA (https://www.ebi.ac.uk/pdbe/pisa/). The service analyzes whether the interface of the components in the crystal is biological assembly or not.
Response: In the original manuscript, we indeed used PDBePISA to perform this analysis. The tetrameric interface, observed in the server, was deemed biological using this server. However, we regretfully did not cite the use of PDBePISA in our original submission. This is corrected in the revised version of our manuscript:

"While crystal packing analysis using the Protein interfaces, surfaces and assemblies (PISA) server [23] suggested several possible assemblies, ranging from heterodimers to dodecamers, only one biological tetrameric assembly consistent with the oligomeric state in solution was detected."

4. No substrate binding site in NgBR
The authors refer Figure 4C to show no pocket in NgBR. However, I am wondering that a solvent accessible pocket might be seen from a different angle.
Probably, some part of the structure in figure 4C might be superposed on the coordination used to draw figure 4A, and the two figures were drawn from the same angle. Correct? Anyway, please describe how the figure is drawn in detail.
Regarding figure 4D, the authors may want to show that several strands and helices of NgBR occupy the substrate binding cavity on DHDDS (text P9 L29-33). If correct, please clearly represent the substrate binding site in DHDDS in Figure 4D.

Response: Thank you for this comment. As the reviewer describes, we first prepared panel a, and then superposed NgBR onto DHDDS and used identical clipping parameters for both panels. The figure legend was modified to better explain how the figure was prepared:
"Clipped surface representation of the heterodimer showing the lack of solvent accessible cavity in NgBR. This view was obtained by superposition of NgBR onto DHDDS as viewed in panel a."
Moreover, we have expanded the legend description to help the reader identify the structural elements of NgBR that prohibit binding of FPP:

"Cartoon representation of a superposition between the cis-prenyltransferase homology domains of DHDDS and NgBR. The secondary structure elements are indicated. Note the tight packing of strands βA, βB, βD and helices α1 and α6, leading to a collapse of the hydrophobic tunnel in NgBR, prohibiting FPP binding."

5. MD simulation (P.10 L8-24)
The authors describe the distance distributions in Figures 5D and 5E in order to show the interactions between 42

and 234, as well as between 42 and 38. However, it sounds nonsense to show the distance distribution between the positively charged heads of K42 and R38, and between the negatively charged carboxylates of E42 and E234. It is not surprising the distance distribution of K42E is wider than that for WT in Figure 5D, because the former does not form attractive interaction. I obtained a similar impression from Figure 5E.
How are the distance distributions between the corresponding Cα atoms? Showing the distance distributions for Cα atoms together with those for the charged group may strengthen the discussion.
Also, how are the B-factor of the amino head of K42 and carboxylate group of E234? They should be reasonably low comparing to those for the surrounding atoms, since the two residues are suggested to form a salt bridge.

Response: We agree with the reviewer that the repulsion between residues with similar charges is indeed trivial. It is completely predictable that K42 will not attract to R38 and that E42 will not attract to E234. However, our main point is that the mutant position E42 forms a new stable salt-bridge with a *specific* catalytic residue. Moreover, E42 does not form a stable salt-bridge with the adjacent R37 or K41 according to our simulations (see also the new Figure S12). We showed the distance distribution for positions 38-42 and 42-234 in both the WT and mutant proteins to help the readers readily identify the differences between the WT and mutant proteins. We now further elaborate our main point in the revised manuscript:

"Specifically, while the WT protein displays a narrow distribution of short distances between positions 42-234, introduction of K42E leads to a wider distribution over longer distances, as expected due to the charge repulsion (Figure 5d). Conversely, while K42E displays a narrow distribution of short distances between position 38-42, these positions display a wider distribution of longer distances in the context of the WT protein, as expected due to their similar charges (Figure 5e). Importantly, despite the presence of adjacent positively charged residues (e.g. R37, K41), E42 forms a stable salt bridge specifically with R38 (Figure S12)."

Importantly, this salt-bridge switch is not expected to perturb the Cα position and such displacement is not required for impairing the enzymatic function. Indeed, in line with your request, we analyzed the Cα distances and revealed that they are stable throughout the simulation between both positions in both the WT and mutant proteins, as shown in the attached plot.

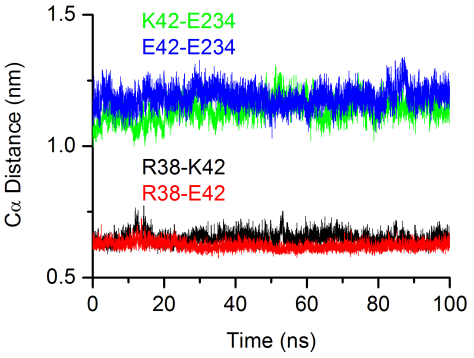

Regarding the B-factors – K42 is adjacent to the substrate coordinating residues R37 and R38 which are highly stabilized by the binding of FPP, and E234 is surrounded by hydrophobic residues which are largely solvent inaccessible and stabilized by adjacent residues, so their B-factors are not exceptionally low compared to their surrounding residues.

6. Labels in the figures
Some texts are too small to read. For example, the labels DHDDS and NgBr in Figure 1A are too small. At least 6 points are recommended except for the super/sub-scripts. Please update ALL figures. It is hard to see the color for fonts with black rims, especially the small labels such as Figure 2. Please consider using the dark color with the same hue, and remove the black rim.

Response: Thank you for this suggestion. We have modified all the figures accordingly.

Minor points

7. Figure 1A

Isoprene unit colored in red should be *cis*-isoprene. Please correct.

Response: Corrected, thank you for your attention.

8. P.8. L.20

The authors refer Figure 3D. Is this correct? If yes, what is βB-α3 linker? By the caption for figure 3D, this figure may represent S2 site, but the sentence describes S1 site. I am confused.

Response: Sorry for the confusion. Indeed, we meant to refer to figure 3c. Thank you.

9. P.11 L3.3

Please show where the helix-turn-helix motif following α7 is in Figure 2.

Response: The helix-turn-helix is now indicated in figure 2a, right panel.

10. P.12 L.17

Normally, a lysine does not attractively interact with arginines. So the sentence "K293* interacts with..." sounds strange. For the reader's convenience, please specify the carboxylate of C- terminal at K293* interact with arginines.

Response: We have modified this sentence according to the reviewer's suggestion:

"Finally, we show that the backbone carboxylate of K293* interacts with the catalytic residues R37 and R85"

11. Units

- A space is required before the degree unit "°C". For example, "16°C" in P.14, L.10 and L.11.
- A space is required before the voltage unit "V" or "kV". For example, "40V", "20V" and "1.8kV" in P.14, L.31-32.
- A space is required before the flow-rate unit "ml/min". For example, "4ml/min" in P.14, L.31.
- Please unify either "h" or "hour" throughout the manuscript. For example, "16-20 h" in P.14, L.12 and "1 hour" in P.15, L.18".
- Please replace "°" into a space at "100°K" in P.15, L.14. - Insert a space in "5Å" in P. 25, L.13.

Response: We made all the suggested corrections.

12. Abbreviations

All abbreviation should be spelled out at their first appearance. For example, "HEPES", "TCEP" in P.14 L17 and L18.

Response: The abbreviations are now spelled out.

13. P.15, L.3. Km may be K_M

Response: Corrected throughout the manuscript, thank you.

14. P.15 L.13. What is NaP?

Response: $NaPO_4$. Corrected.

15. P16. L.4
Please remove underline between the "(WT and mutant)" and "The resulting".

Response: Removed, thank you.

16. Table 1
- Please show the resolution range for the full dataset (not only in the parenthesis).
- "R" of "R_{meas}", "R_{work}", "R_{free}", "CC" of "$CC_{1/2}$", "I" of Mean $I/\sigma(I)$ should be italicized.
- Please add the unit (Å) for the "Resolution range" line at the "Refinement statistics".
- Please add the units (Å, °) for the "RMSD (bond lengths/angles)" lines.
- Please describe what programs/servers are used to perform the ramachandran analysis with the proper citations. Also, please provide the rate of favorite/acceptable residues by the ramachandran analysis.

Response: We made all the suggested changes, thank you. The Ramachandran analysis was performed using MolProbity, as now cited in the methods section:

"Ramachandran analysis was performed using MolProbity [38]."

17. Supplementary Figure S3
20 mM FPP and 100 mM IPP may be typo. mM may be μM.

Response: Corrected, thank you.

18. Supplementary Figure S4
What is the difference between the panels A and B? I mean, why do the authors separate the 8 SEC-MALS analyses into 2 parts? Is there any difference between the red (S4A) and green (S4B) curves? Also, I am wondering why the molecular weight is shown in blue in Figure S3A. If there are no experimental difference, drawn in the same color is preferred.

Response: According to the reviewer's suggestion, the SEC-MALS analyses are no longer divided into panels and the molecular weight curve is colored red throughout, as in Figure 1.

Reviewer #4:

In this manuscript, high-resolution crystal structure has been determined for the human cis-prenyltransferase (hcis-PT), which is an essential enzyme for protein N-clycosylation and implicated in human diseases including autosomal recessive retinitis pigments (arRP), epilepsy, neurological impairments and hearing deficit. Disease mutations of hcis-PT around the active site have been investigated through combined experiments and molecular dynamics (MD) simulations, which provide intriguing mechanistic insights into the enzyme dysfunction in arRP. Overall, the manuscript is well written. The results are supported by the presented data. For the MD simulations, the manuscript can be still improved with the following major suggestions:

Response: We would like to thank the reviewer for the thoughtful overview and comments.

1. Three 100 ns MD simulations have been performed on both the WT and K42E mutant of hcis-PT. But why are results shown for only one of the three simulations in Fig. 5 and Fig. S8? All the simulations should be analyzed carefully. The results need to be presented for individual simulations and compared to evaluate the simulation convergence, especially given that 100 ns seems pretty short with state-the-of-art MD simulations.

Response: Fig. 5 and Fig. S8 (now Fig. S11) represent the average of the three simulations. In line with the reviewer's suggestions, the figure legends were modified to highlight that the data are presented as averages:

*"**(d)** Distance distribution between positions 42 and 234, measured between the charge centers of E234 and K42 (light blue) or E42 (light orange). Inset: Average distances along the simulations trajectories. **(e)** Distance distribution between positions 38 and 42, measured between the charge centers of R38 and K42 (light blue) or E42 (light orange). Inset: Average distances along the simulations trajectories. **(f)** Active site residues within 5 Å of the crystallized substrates are colored cyan and shown as sticks. **(g)** Average RMSD of the active site residues indicated in panel F along the simulations trajectory."*

SD values are not shown as they pose a challenge for data visualization by the reader. Instead, we now provide the data from individual simulations in Figure S11a-c.

2. Related to the above point, in Fig. S8 it may help to plot the averages and standard deviations (error bars) of the residue RMSFs that can be calculated from three individual simulations of each system.

Response: We added error bars to the RMSF plots (Figure S11d). Thank you.

3. In Fig. 5D, the distance was "measured between the carboxylate oxygens of E234 and the side-chain nitrogen (Nz) of K42 (light blue) or the carboxylate oxygens of E42 (light orange)." This may lead to multiple atom distances given the description. Do the authors calculate the minimum atom distance of their measurements between two interested residues? This is not clear and needs clarification, similarly for the distance plots in Fig. 5E. Alternatively, the authors can probably use charge centers of the basic and acidic residue side chains to avoid such confusion and fluctuations of the distance plots.

Response: Thank you for this suggestion! We have re-calculated the distances according to the charge centers and updated the figures (5 and S11) and text accordingly. Of note, this re-analysis did not affect our

conclusions.

4. In addition to the RMSD plot in Fig. 5G, it would help to compare the simulation final structure of the K42E mutant with the WT, especially for the enzyme active site.

Response: In line with your suggestion, we added a view of the active site and important catalytic residues in the final structures from representative simulations of each protein to the new Figure S12.

REVIEWER COMMENTS</B>

Reviewer #1 (Remarks to the Author):

Manuscript by Bar-El et al., addresses key structural and mechanistic aspects of h cis-iPTase complex. This work is critical in advancing our understanding of congenital disorders pertaining to dolichol synthesis. The reviewer finds this manuscript acceptable for publication pending two minor edits.
1. In Line 67, the statement ending with reference#15 should be followed with a statement regarding the lack of N-glycosylation or mannose incorporation defects in patient samples from K42E and severe stop/nonsense mutations (ref#12-16). This needs to be explicitly mentioned.

2. The authors have added a new Figure S6, with the structure of DHDDS color-coded according to the conservation score of each residue, along with the sequence alignment for several species. This figure seems very helpful in understanding the structure-function conservation of the h cis—Ptase in relation to other species. The figure will benefit by providing the conservation score for K42E and T206A point mutations in the figure legend (and if possible also highlight those residues in the structure).

Reviewer #2 (Remarks to the Author):

All questions raised have been properly addressed. Now the manuscript has become suitable for publication.

Reviewer #3 (Remarks to the Author):

Almost all parts of the manuscript are adequately revised except for the following points. The manuscript is recommended to be accepted after the following minor revisions.

The following comment # correspond to the numbers of the previous my (Reviewer #3) comments.

Comment 1 (Crystal structure)
The authors provided electron density map as Fig S4. Although they said the maps are "2mFo-DFc", I guess they are "mFo-DFc" as they said in the response letter. If the maps are 2mFo-DFc omit electron density map, there are several questionable points.

Comments 2 (SDS-PAGE)
"Molecular Weight (kDa)" should be "Molecular Mass /kDa" in Figure 1B.

Comment 6 (Labels in the figures)
Because they enlarged the font size and removed the rim, it is much easy to understand the figure. One more. Please consider darker colors (with the same hue) to represent labels. If they have already tried the dark color and they think the present colors are the best, no change is required.

Reviewer #4 (Remarks to the Author):

The authors have addressed most of my previous comments, except the following concerns:

1. In Fig. 5, are the "average distances" and "average RMSD" averaged over three different simulations? Given the variations observed in Fig. S11 among the different simulations, it may not be valid / meaningful to plot these averages. In this context, the distance distributions (with statistics

collected from all three simulations combined) are still valid.

2. Related to the above point, in Fig. S11C RMSD of active site residues in one of the K42E mutant simulations (red) was shown to be similar and even slightly smaller than that of the WT simulations. This suggests that the K42E mutant active site could be also stable. What's special about this simulation? Further analysis and discussion are needed.

Furthermore, it could be equally relevant if not more to compare closely the fluctuations / RMSFs of active site residues in the K42E mutant and WT.

3. In Fig. S12, how were the "representative" simulations selected, especially for the K42E mutant?

Sackler Faculty of Medicine
Tel Aviv University

Laboratory of structural physiology | Dept. of Physiology and Pharmacology | Tel Aviv, Israel 6997801 | office 972-3-6409261 | fax 972-3-6409894

Reviewer #1:

Manuscript by Bar-El et al., addresses key structural and mechanistic aspects of h cis-iPTase complex. This work is critical in advancing our understanding of congenital disorders pertaining to dolichol synthesis. The reviewer finds this manuscript acceptable for publication pending two minor edits.

Response: We thank reviewer #1 for the thorough assessment and finding our revised manuscript acceptable for publication.

1. In Line 67, the statement ending with reference#15 should be followed with a statement regarding the lack of N-glycosylation or mannose incorporation defects in patient samples from K42E and severe stop/nonsense mutations (ref#12-16). This needs to be explicitly mentioned.

Response: In references #12-13, the glycosylation phenotype was not assessed. Reference #14 describes "normal glycosylation assays" in two patients, but such studies were not performed for all the developmental epileptic encephalopathy patients in this cohort. In reference #15, describing the severe mutations, hypoglycosylation of serum proteins and reduced dolichol-phosphate levels were detected. Moreover, assays using the patient's fibroblasts initially revealed a hypo-glycosylation phenotype, but this phenotype gradually dissipated with the passage number, for a reason they could not resolve. Reference #16 describes the R290H mutation in NgBR, which causes glycosylation defects, as we describe in our manuscript. To further address these complex genotype-phenotype relationships, we have modified the revised version:

"For example, while the missense mutation in the RxG motif of NgBR led to reduced glycosylation in patients fibroblasts [16] and the patient suffering from the fatal glycosylation disorder displayed hypoglycosylation of serum glycoproteins [15], the arRP mutation in DHDDS does not seem to have any significant effect on glycosylation in a knock-in mouse model [18,19] and some patients with DHDDS-related developmental epileptic encephalopathy display normal glycosylation assay results [14]." (Introduction, Page 4).

We believe that further discussion of these studies is beyond the scope of our manuscript.

2. The authors have added a new Figure S6, with the structure of DHDDS color-coded according to the conservation score of each residue, along with the sequence alignment for several species. This figure seems very helpful in understanding the structure-function conservation of the h cis—Ptase in relation to other species. The figure will benefit by providing the conservation score for K42E and T206A point mutations in the figure legend (and if possible also highlight those residues in the structure).

Response: Thank you for this suggestion. As we do not address T206 in this manuscript we decided to add only the conservation score of K42 to the figure legend.

Laboratory of structural physiology | Dept. of Physiology and Pharmacology | Tel Aviv, Israel 6997801 | office 972-3-6409261 | fax 972-3-6409894

Reviewer #2:

All questions raised have been properly addressed. Now the manuscript has become suitable for publication.

Response: We thank reviewer #2 for the time and effort invested to improve our manuscript.

Sackler Faculty of Medicine
Tel Aviv University

Laboratory of structural physiology | Dept. of Physiology and Pharmacology | Tel Aviv, Israel 6997801 | office 972-3-6409261 | fax 972-3-6409894

Reviewer #3:

Almost all parts of the manuscript are adequately revised except for the following points. The manuscript is recommended to be accepted after the following minor revisions.

Response: We thank reviewer #3 for the constructive and helpful critique, and for finding our revised manuscript acceptable for publication following the proposed changes.

The following comment # correspond to the numbers of the previous my (Reviewer #3) comments.

Comment 1 (Crystal structure)
The authors provided electron density map as Fig S4. Although they said the maps are "2mFo-DFc", I guess they are "mFo-DFc" as they said in the response letter. If the maps are 2mFo-DFc omit electron density map, there are several questionable points.

Response: Corrected, thank you.

Comments 2 (SDS-PAGE)
"Molecular Weight (kDa)" should be "Molecular Mass /kDa" in Figure 1B.

Response: Corrected in Fig. 1 as well as in Fig. S3 and S7. Thank you.

Comment 6 (Labels in the figures)
Because they enlarged the font size and removed the rim, it is much easy to understand the figure. One more. Please consider darker colors (with the same hue) to represent labels. If they have already tried the dark color and they think the present colors are the best, no change is required.

Response: We did try a darker hue according to your previous suggestion but thought that the current color scheme is better.

Sackler Faculty of Medicine
Tel Aviv University

Laboratory of structural physiology | Dept. of Physiology and Pharmacology | Tel Aviv, Israel 6997801 | office 972-3-6409261 | fax 972-3-6409894

Reviewer #4:

The authors have addressed most of my previous comments, except the following concerns:

1. In Fig. 5, are the "average distances" and "average RMSD" averaged over three different simulations? Given the variations observed in Fig. S11 among the different simulations, it may not be valid / meaningful to plot these averages. In this context, the distance distributions (with statistics collected from all three simulations combined) are still valid.

Response: Yes, the plots represent the averages of all the simulations. We have removed the insets of the average distances along the simulations from panels d and e. Moreover, in line with the next comment, and replaced the RMSD plot with the RMSF of the relevant residues.

2. Related to the above point, in Fig. S11C RMSD of active site residues in one of the K42E mutant simulations (red) was shown to be similar and even slightly smaller than that of the WT simulations. This suggests that the K42E mutant active site could be also stable. What's special about this simulation? Further analysis and discussion are needed.

Furthermore, it could be equally relevant if not more to compare closely the fluctuations / RMSFs of active site residues in the K42E mutant and WT.

Response: Thank you for this comment. We would like to highlight that the main finding of our MD simulations is the formation of the R38-E42 bridge as a pathological mechanism for hindered catalytic activity. The observation of active site destabilization came to us as a surprise, but due to its potential significance we mainly addressed it in the discussion and were careful in its interpretation:

"Specifically, we suggest that the globally enhanced dynamics of the active site may result in weakening of the association with long-chain products, leading to their premature release. Future studies are needed to determine whether altered substrate interaction, either through direct or indirect mechanisms, converge into a similar enhancement of active site dynamics, resulting in a common outcome of products length shortening, along with the reduction in catalytic activity (Fig. 3f, 5a)." (Discussion, page 17)

However, to alleviate the concern you raised, we performed an additional simulation for the mutant complex. In this simulation, the RMSD of the active site is also increased compared to the WT complex (Supplementary Fig. S11). Of course, the formation of the R38-E42 is clearly observed in this simulation as in the other replicates (Supplementary Fig. S11). Moreover, we modified Fig. 5 to show the RMSF values of active site residues instead of the average RMSD along the simulation. Indeed, the RMSF values of the active site residues (within 5Å from the bound substrates) are consistently slightly higher in the mutant compared to the WT protein (Fig. 5f). Finally, in line with your comment and to prevent any potential misrepresentation of our findings, we have further revised the results and discussion section to highlight the differences between the simulations:

"In addition, we examined the effect of K42E on the dynamics of the entire active site by monitoring active site residues within 5Å of the crystallized substrates (Fig. 5f). We noticed that the within the timeframe of the simulations, the K42E mutation results in active site destabilization, including the C-terminus of NgBR, as reflected

by the overall increase in RMSD (representing the deviation of the coordinates relative to the initial state of the simulation) in three of four simulations (Supplementary Fig. 11) and by the overall increase in RMSF (representing the fluctuation of the residue side chain relative to its average position along the simulation) (Fig. 5f, Supplementary Fig. 11). These results support the notion that K42E underlies the formation of aberrant polar networks, hindering the capacity of active site residues to bind substrates, thereby reducing the catalytic activity". (Results, page 14)

"Interestingly, additional to this local structural alteration, we also observed a tendency for global increase in active site dynamics, including the C-terminus of NgBR (Fig. 5f, Supplementary Fig. 11). Inspection of the active site residues RMSD along the trajectories of the individual simulations revealed destabilization in three of the four replicates (Supplementary Fig. 11c) and the mean RMSF values of these residues are consistently slightly higher in the mutant compared to the WT complex (Fig. 5f)." (Discussion, page 17)

3. In Fig. S12, how were the "representative" simulations selected, especially for the K42E mutant?

Response: In the previous version, we chose to show the last state from the last simulation. However, in line with your comment, we have updated Supplementary Fig. 12 to include the final state of each simulation. As can be inferred from the R38-E42 distance plots (Supplementary Fig. 11), this salt-bridge is observed in the final conformations of two simulations. We would like to underscore that we performed cluster analysis and the conformations shown in Fig. 5 represent the most abundant cluster (this point has been clarified in the revised figure legend), consistent with the R38-E42 distance plot showing close proximity between their charge center during most of the simulation.

REVIEWERS' COMMENTS

Reviewer #4 (Remarks to the Author):

The authors have addressed my comments adequately. I would recommend publication of the manuscript.

Sackler Faculty of Medicine
Tel Aviv University

Laboratory of structural physiology | Dept. of Physiology and Pharmacology | Tel Aviv, Israel 6997801 | office 972-3-6409261 | fax 972-3-6409894

Reviewer #4:

Response: We thank reviewer #4 for the time and effort invested to improve our manuscript.
